# Clinical outcomes of glucagon-like peptide-1 receptor agonist therapy in kidney transplant recipients: a systematic review and meta-analysis

**DOI:** 10.1093/ckj/sfag105

**Published:** 2026-03-26

**Authors:** Mehmet Kanbay, Sama Mahmoud Abdel-Rahman, Mustafa Guldan, Lasin Ozbek, Nur I Genc, Ahmet B Ak, Adrian Covic, Hayri K Goren

**Affiliations:** Division of Nephrology, Department of Internal Medicine, Koc University School of Medicine, Istanbul, Türkiye; Koç University School of Medicine, Istanbul, Türkiye; Koç University School of Medicine, Istanbul, Türkiye; Koç University School of Medicine, Istanbul, Türkiye; Department of Internal Medicine, Koc University School of Medicine, Istanbul, Türkiye; Department of Internal Medicine, Koc University School of Medicine, Istanbul, Türkiye; Nephrology Clinic, Dialysis and Renal Transplant Center, “C.I. Parhon” University Hospital and “Grigore T. Popa” University of Medicine, Iasi, Romania; Department of Internal Medicine, Koc University School of Medicine, Istanbul, Türkiye

**Keywords:** glucagon-like peptide-1 receptor agonists, kidney transplantation, liraglutide, semaglutide, tacrolimus

## Abstract

**Background:**

Metabolic complications after kidney transplantation (KT) significantly affect graft and patient survival. Glucagon-like peptide-1 receptor agonists (GLP-1RAs) offer cardio-renal benefits in the general population, but evidence in KT recipients remains limited.

**Methods:**

We conducted a systematic review and meta-analysis following Preferred Reporting Items for Systematic Reviews and Meta-Analyses 2020 guidelines (PROSPERO: CRD420251153352). PubMed, Scopus, Web of Science, Ovid MEDLINE, and Cochrane Library were searched up to September 2025 for studies evaluating GLP-1RA therapy in adult KT recipients. Random-effects models pooled outcomes for metabolic, renal, cardiovascular, and safety endpoints.

**Results:**

Seventeen studies (*n* = 54 680 KT recipients) were included. GLP-1RAs use significantly reduced all-cause mortality (HR 0.53, 95% CI 0.36–0.79, *k* = 4) and major adverse cardiovascular events (OR 0.55, 95% CI 0.47–0.66, *k* = 3). Estimated glomerular filtration rate (eGFR) remained stable at 3 months, improved at 6 months (+1.99 ml/min/1.73 m², 95% CI 0.52–3.47, *k* = 5) and 12 months (+2.24 ml/min/1.73 m², 95% CI 0.02–4.46, *k* = 6), and was preserved at 24 months. GLP-1RAs lowered HbA1c (MD −0.54%, 95% CI −0.89 to −0.19, *k* = 13) and body mass index (SMD −0.32, 95% CI −0.49 to −0.15, *k* = 12) from baseline, with parallel reductions in insulin requirement and urinary albumin excretion. Tacrolimus levels were unaffected at 6 months and modestly decreased at 1 year without compromising graft function. Adverse events were mainly mild gastrointestinal intolerance (10%–20%), with rare discontinuations and no increased risk of hypoglycemia, pancreatitis, or infections.

**Conclusion:**

GLP-1RAs are associated with improved glycemic control, weight, and cardiovascular outcomes, with no consistent signal of adverse effects on graft stability and immunosuppressive balance. GLP-1RA integration into individualized post-transplant care may be considered for patients with diabetes or metabolic syndrome, with close clinical monitoring.

## INTRODUCTION

Kidney transplantation (KT) remains the optimal therapeutic intervention for patients with end-stage kidney disease, offering substantial improvements in survival and quality of life compared with maintenance dialysis. Despite these advantages, long-term outcomes following KT are often compromised by the high prevalence of metabolic complications, including post-transplant diabetes mellitus (PTDM), obesity, dyslipidemia, and cardiovascular disease [1]. These conditions not only increase the risk of graft loss and cardiovascular mortality but may also pose challenges in optimizing immunosuppressive therapy and minimizing drug interactions [1]. Factors such as chronic corticosteroid exposure, calcineurin inhibitor-induced insulin resistance, and post-transplant weight gain may also contribute to this complex metabolic milieu [2].

Glucagon-like peptide-1 receptor agonists (GLP-1RAs) have emerged as an important class of agents for managing type 2 diabetes mellitus and obesity. GLP-1RAs potentiate glucose-dependent insulin secretion, suppress glucagon release, delay gastric emptying, and promote satiety and weight reduction. Large randomized controlled trials have demonstrated significant cardiovascular and renal benefits, including reduced major adverse cardiovascular events (MACE) and slower progression of kidney disease [3]. Consequently, GLP-1RAs are now recommended as first- or second-line therapy for patients with type 2 diabetes and established cardiovascular or renal risk.

However, application of GLP-1RAs in kidney transplant recipients has historically been cautious due to concerns about potential drug–drug interactions with immunosuppressive agents (notably tacrolimus and cyclosporin), altered pharmacokinetics with variable graft function, and limited transplant-specific evidence. Additionally, gastrointestinal side effects, such as nausea, vomiting, and reduced appetite, may complicate the use of critical immunosuppressants, raising concerns about maintaining therapeutic drug levels and graft stability. Early transplant-focused reviews framed this hesitancy and called for randomized controlled trials (RCTs) [4]. Since then, observational data, systematic reviews, and meta-analyses suggest that GLP-1RAs may provide favorable metabolic and renal effects in KT recipients, including improvements in glycemic control, body weight, and possibly preservation of graft function while some studies have also reported neutral or beneficial effects on tacrolimus levels [5–7], though findings remain inconsistent and limited by small sample sizes, retrospective designs, and heterogeneous patient populations. Importantly, while GLP-1RA RCTs in KT recipients are still lacking, transplant-specific randomized studies are ongoing (e.g. semaglutide to prevent PTDM: NCT06913023), and an RCT program exists for sodium-glucose cotransporter 2 inhibitors (SGLT2i) in KT (NCT04965935), reflecting rapidly evolving evidence. A recent systematic review and meta-analysis summarized early observational evidence in kidney transplant recipients (nine cohort studies; *n* = 338) and reported improvements in glycemic control, weight, and proteinuria with no significant change in tacrolimus levels, although hard outcomes remained sparsely reported [7]. However, additional data have since emerged, including larger real-world cohorts and comparative analyses, warranting an updated synthesis.

Therefore, this systematic review and meta-analysis aimed to evaluate the safety, efficacy, and clinical outcomes associated with GLP-1RA use in kidney transplant recipients. Specifically, we assessed their effects on metabolic parameters (including glycemic indices and body weight), renal allograft function, immunosuppressive therapy, and adverse events such as gastrointestinal intolerance, infection, and drug discontinuation. By integrating findings across existing studies, this analysis seeks to clarify the clinical utility of GLP-1RAs in this unique and high-risk population.

## MATERIALS AND METHODS

This systematic review and meta-analysis were reported in accordance with the Preferred Reporting Items for Systematic Reviews and Meta-Analyses (PRISMA) 2020 checklist (Supplementary Table S1). The study protocol was prospectively registered in the International Prospective Register of Systematic Reviews (PROSPERO) under the registration number of CRD420251153352.

### Search strategy

A systematic search of PubMed, Scopus, Web of Science, Ovid MEDLINE, and the Cochrane Library was conducted from database inception to 29 September 2025. Search terms combined keywords and MeSH terms related to KT, GLP-1 receptor agonists (liraglutide, semaglutide, dulaglutide, etc.), and multi-receptor agonists such as tirzepatide (GIP/GLP-1 co-agonist) and retatrutide (GIP/GLP-1/glucagon tri-agonist). The full search strategy for each database is provided in Supplementary Table S2. Reference lists of all eligible studies and relevant reviews were also screened for additional records.

### Eligibility criteria

Studies were included if they met the following criteria:

1. Population: Adult kidney transplant recipients (≥18 years).
2. Intervention: Treatment with any GLP-1 receptor agonists, and multi-receptor agonists, regardless of dose or formulation.
3. Comparator: Placebo, other glucose-lowering therapy, or usual care; single-arm studies were also eligible for safety and tolerability outcomes.
4. Outcomes: At least one of the following was reported: (i) immunosuppressive drug levels; (ii) graft rejection or failure; (iii) timing of GLP-1RA initiation post-transplant; (iv) hospitalization; (v) adverse events or discontinuation due to intolerance; (vi) cardiovascular outcomes; (vii) infections; (viii) weight change or weight control; (ix) glycemic control (HbA1c, fasting glucose); or (x) development of post-transplant diabetes mellitus.

Exclusion criteria included reviews, editorials, conference abstracts without extractable data, animal/*in vitro* studies, case reports, and case series including fewer than or equal to five patients.

### Study selection and data extraction

Two reviewers independently screened titles and abstracts for relevance, followed by a full-text review of potentially eligible studies. Discrepancies were resolved through discussion or consultation with a third reviewer. Data were extracted independently by two reviewers using a standardized form. Extracted information included: first author, publication year, country, study design, sample size, follow-up duration, patient demographics, immunosuppressive regimen, GLP-1RA agent and dosage, comparator, and reported outcomes.

### Statistical analysis

We used a random-effects model to pool effect estimates, acknowledging anticipated clinical heterogeneity. For dichotomous outcomes, results were summarized as odds ratios (ORs) with 95% confidence intervals (CIs); for time-to-event outcomes, hazard ratios (HRs) were synthesized; and for continuous outcomes, mean differences (MDs) or standardized mean differences (SMDs) were calculated based on measurement scales. Statistical heterogeneity was assessed with the Chi² test and quantified using *I*². Whenever a concurrent comparator group was available, we prioritized between-group meta-analyses. Within-group pre–post meta-analyses (baseline to follow-up) were performed only when comparative data were not reported or could not be computed from published aggregate data, and these within-group results were interpreted as descriptive rather than causal.

Subgroup analyses based on follow-up duration were performed only for outcomes where follow-up time was not already inherent to the reported effect estimates. Outcomes already stratified by fixed time points (e.g. eGFR at 3, 6, and 12 months) or reported uniformly at a single time point (e.g. HbA1c and BMI at 12 months) were not subgrouped by follow-up. For analyses where follow-up-based subgrouping was not applicable, heterogeneity was instead explored using leave-one-out sensitivity analysis by iteratively removing individual studies to assess the stability of pooled effects. We used random-effects meta-analysis with between-study variance estimated using the restricted maximum likelihood (REML) method. Because several outcomes included a small number of studies (often 2–6), we conducted sensitivity analyses comparing random-effects results with fixed-effect models to evaluate robustness to meta-analytic assumptions.

Potential publication bias was evaluated through visual inspection of funnel plots and Egger’s regression test only when ≥10 studies were available for a given outcome. All analyses were conducted in Review Manager (RevMan 5.3).

### Risk of bias assessment

The Risk of Bias in Non-randomized Studies of Interventions (ROBINS-I) tool was used to assess the quality of the included studies (Supplementary Tables S3), while the Newcastle–Ottawa scale (NOS) was used to assess the quality of published abstracts (Supplementary Table S4). Two reviewers independently evaluated the risk of bias, and any discrepancies were resolved by consensus with a third reviewer.

### Certainty of evidence

The certainty of evidence for each outcome was evaluated using the Grading of Recommendations, Assessment, Development, and Evaluation (GRADE) approach. Because all included studies were retrospective cohort designs, the initial certainty rating started at “low” and was downgraded or upgraded based on five domains: risk of bias, inconsistency, indirectness, imprecision, and publication bias. Each outcome was then categorized as high, moderate, low, or very low certainty, as summarized in Supplementary Table S5.

## RESULTS

**Figure 1:**
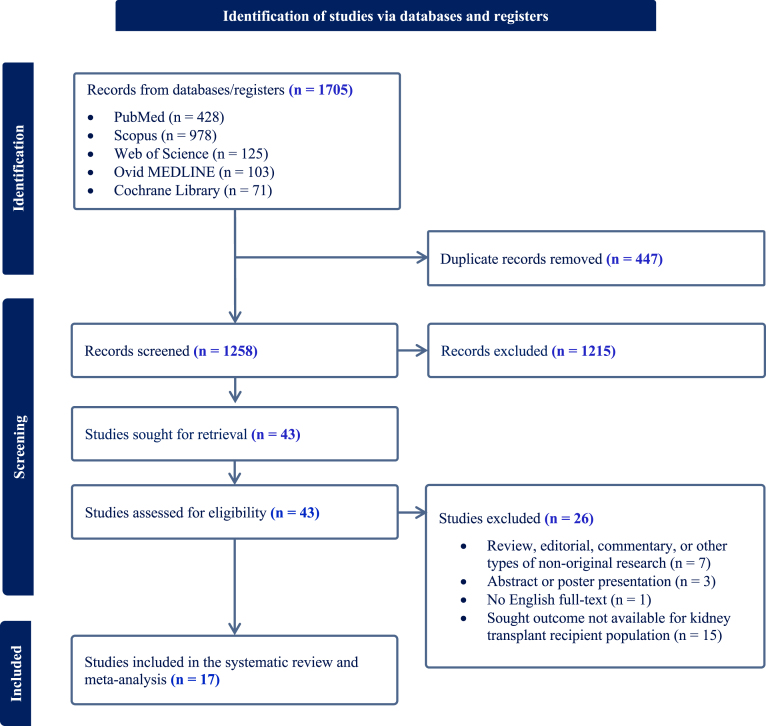
PRISMA flow diagram of the study selection process.

A total of 1705 records were identified. After removing 447 duplicates, 1258 studies were screened, and full texts were assessed for eligibility. Seventeen studies met the inclusion criteria and were included in the final analysis (Fig. 1) [6, 8–22, 23]. Overall, the included studies were mainly observational, conducted across multiple regions with sample sizes ranging from 7 to 3297 GLP1-RA users. Across studies reporting these data, the mean age generally fell in the mid-50s to early-60s and the proportion of male participants ranged from ∼35% to 74%. GLP-1RA exposure was frequently mixed within cohorts, with dulaglutide, liraglutide, and semaglutide most commonly used, and follow-up durations ranged from 6 months to 3.1 years. Where reported, maintenance immunosuppression was typically calcineurin inhibitor-based with corticosteroids and often mycophenolate. A summary of main characteristics of the included studies is provided in Table 1.

**Table 1:** Included studies reporting the use of GLP-1RA in kidney transplant recipients.

First author, year	Study design	Country	Population description	Sample size	Investigated medications	Male (%)	Mean age ± SD (years)	Living donor (%)	Follow-up (months or years)	Maintenance immunosuppression
Diker Cohen *et al*., 2025 [6]	RC	Israel	KTR with pre- or PTDM	272 in total: 136 GLP-1RAs,136 controls	Dulaglutide (52%),liraglutide (29%),semaglutide (19%)	69%	58.3 ± 11.0	NA	3.1 years	NA
Freitas *et al*., 2024 [8]	RC	Portugal	KTR with or without DM	64	GLP1-RA (NA)	62.5%	60.1 ± 12.1	19%	20 months	CNI (93%), mycophenolate mofetil (83%), and steroids (90%).
González *et al*., 2021 [31]	RC	Spain	KTR with or without DM	13	Semaglutide (54%),liraglutide (31%),dulaglutide (15%),or SGLT2i	35.3%	54	NA	12 months	No information other than tacrolimus and steroids
Kahwaji *et al*., 2024 [10]	RC	USA	KTR with DM	185	Liraglutide (61%),semaglutide (39%)	44%	56 ± 11	NA	6 months	No information other than tacrolimus
Kim *et al*., 2021 [11]	RC	South Korea	KTR with DM	37	Dulaglutide (100%)	48.6%	54.8 ± 8.5	NA	6 months	Tacrolimus, cyclosporine, steroids
Kukla *et al*., 2020 [12]	RC	USA	KTR with/without pre- or PTDM	17	Liraglutide (82%),exenatide (6%),dulaglutide (12%)	65%	51.8	77%	12months	Tacrolimus, everolimus, mycophenolate mofetil, steroids
Lin *et al*., 2025 [13]	RC	Multi-country (21 countries)	KTR with DM	35 488 in total: 3297 GLP-1RAs,32 023 controls	GLP1-RA (NA)	57.7%	57.7 ± 12.2	NA	2.5 years	Tacrolimus, cyclosporine, steroids
Liou *et al*., 2018 [14]	RC	Taiwan	KTR	7	Liraglutide (100%)	NA	NA	NA	19.4 ± 7.6months	NA
Mahmoud *et al*., 2023 [15]	RC	Kuwait	KTR with DM	209 in total: 98 SGLT2i, 41 GLP1-RA70 controls	Dulaglutide,liraglutide,and SGLT2i	56.1%	53	73.17%	12 months	Tacrolimus, cyclosporin
Mahzari *et al*., 2024 [5]	RC	Saudi Arabia	KTR with pre- or PTDM	39	Semaglutide (100%)	74%	54 ± 9	NA	18 months	Tacrolimus, cyclosporine, steroids, Mycophenolate mofetil
Mallik *et al*., 2023 [17]	RC	UK	KTR with pre- or PTDM	23	Dulaglutide (74%),liraglutide (26%)	65.2%	56.5	NA	26.5 ± 16.5 months	Tacrolimus, cyclosporin
Orandi *et al*., 2025 [18]	RC	USA	KTR with DM	18 016	GLP1-RA (NA) (15.9%)	64,30%	59.7	13,20%	12 months	NA
Sahi *et al*., 2025 [19]	RC	USA	KTR with DM	2171 in total: 77 GLP-1RAs,2094 controls	Dulaglutide (46.8%), semaglutide (29.8%),liraglutide (23.4%)	61%	57.9 ± 9.5	NA	12 months	Tacrolimus, mycophenolate mofetil, steroids,
Sato *et al*., 2023 [28]	RC	Japan	KTR with DM	146 in total: 73GLP-1RAs, 73controls	Liraglutide,dulaglutide,exenatide,lixisenatide	65.8%	55	NA	24 months	Mycophenolate Mofetil or everolimus,tacrolimus orcyclosporine, prednisone
Vigara *et al*., 2022 [21]	RC	Spain	KTR with DM	40	Semaglutide (48%),liraglutide (32%), dulaglutide (20%)	56.2%	61.6	NA	12 months	Tacrolimus, mycophenolate, everolimus, steroids
Vigara *et al*., 2024 [27]	RC	Spain	KTR with DM	96	Semaglutide (67%),liraglutide (21%),dulaglutide (12%)	54%	61.6 ± 9.7	56.2%	12 months	Tacrolimus, mycophenolate mofetil, mycophenolate sodium, everolimus, cyclosporine, prednisone;
Zelada *et al*., 2025 [23]	RC	USA	KTR with DM	50 in total:25 GLP-1RAs,25 controls	Dulaglutide (8%), semaglutide (80%),liraglutide (12%)	64%	56	NA	12 months	CNI, mTORi, glucocorticoids

GLP1-RA, glucagon peptide 1 receptor agonist; RC, retrospective cohort; KTR, kidney transplant recipients; LRT; liver transplant recipients; DM, diabetes mellitus; PTDM: post-transplant diabetes mellitus; SD, standard deviation; NA: no data available; USA, United States of America; UK, United Kingdom, CNI: calcineurin inhibitors.

**1. Mortality**

**Figure 2:**
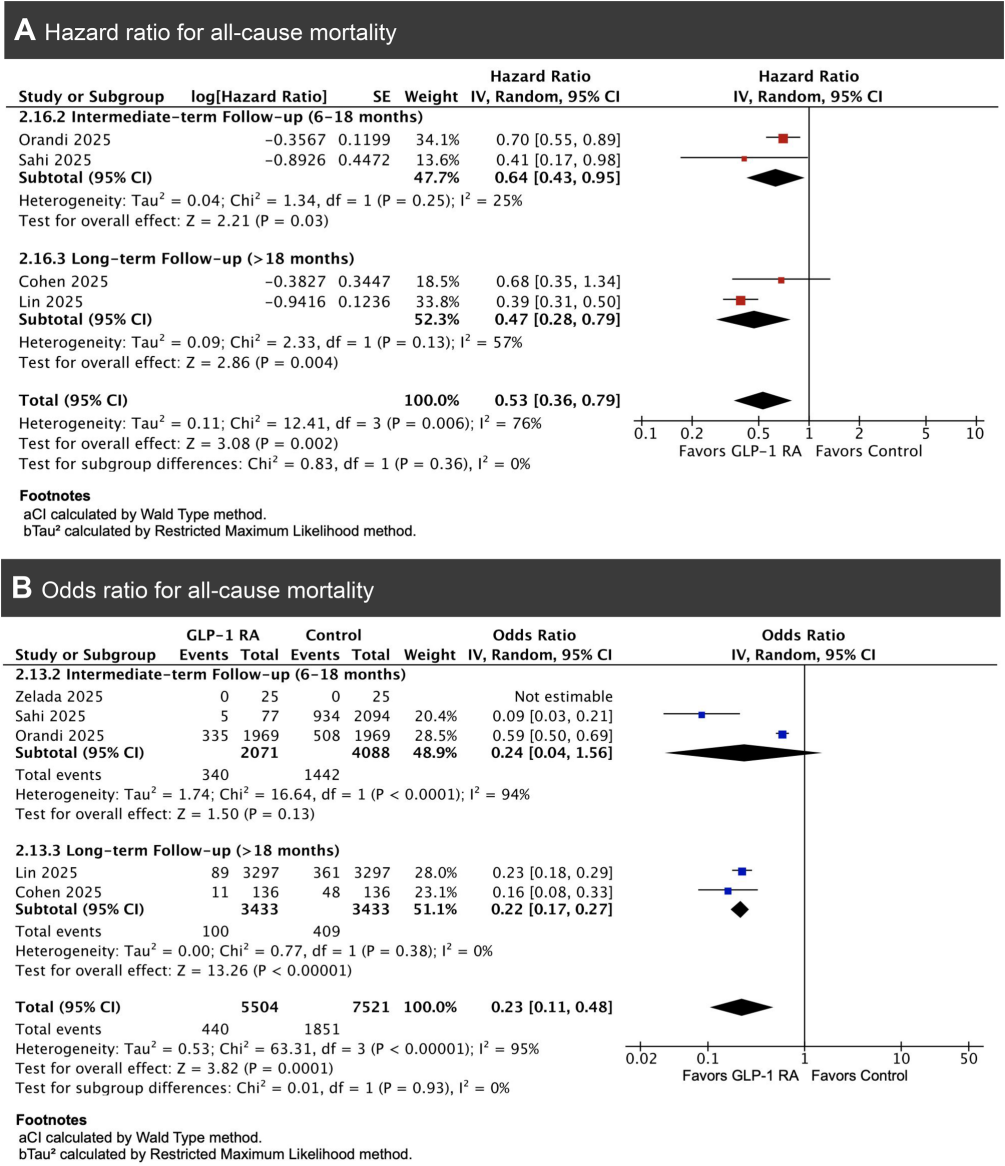
Forest plot for all-cause mortality among kidney transplant recipients treated with GLP-1 receptor agonists compared with non-users: (A) pooled HR and (B) pooled OR using events and totals.

Pooled analysis of HRs from four studies demonstrated a significant reduction in all-cause mortality with GLP-1RA use (HR 0.53, 95% CI 0.36–0.79, *P* = .002), with moderate heterogeneity (*I*² = 76%) [6, 24–26]. Subgroup analyses by follow-up duration were performed to explore heterogeneity. The test for subgroup differences was not significant (χ² = 0.83, df = 1, *P* = .36, *I*² = 0%), although heterogeneity declined within subgroups (*I*² = 25% and 57%) (Fig. 2A). A complementary meta-analysis using event counts from five studies yielded consistent results (OR 0.23, 95% CI 0.11–0.48, *P* = .0001) [6, 13, 18, 19, 23]. The test for subgroup differences was not significant (χ² = 0.62, df = 1, *P* = .43, *I*² = 0%), and heterogeneity remained high overall (*I*² = 95%) but decreased within subgroups (94% and 0%, respectively) (Fig. 2B).

**2. Major cardiovascular events**

**Figure 3:**
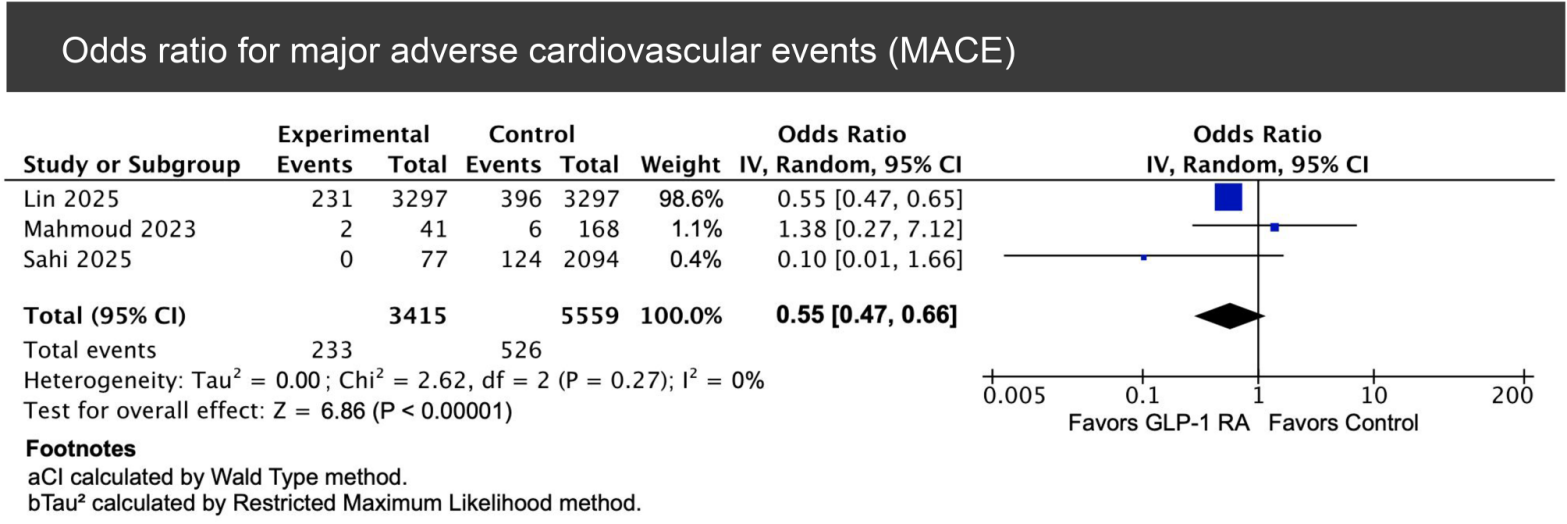
Forest plot of the pooled OR for MACE among GLP-1RA users vs. non-users.

In the pooled analysis of three studies [15, 24, 26], GLP-1RA treatment was associated with a significantly reduced risk of MACE (233 vs. 526 events; OR 0.55, 95% CI 0.47–0.66, *P* < .00001), with no evidence of heterogeneity (*I*² = 0%) (Fig. 3).

**3. Estimated glomerular filtration rate comparison between GLP-1RA users versus controls at 12 months**

**Figure 4:**
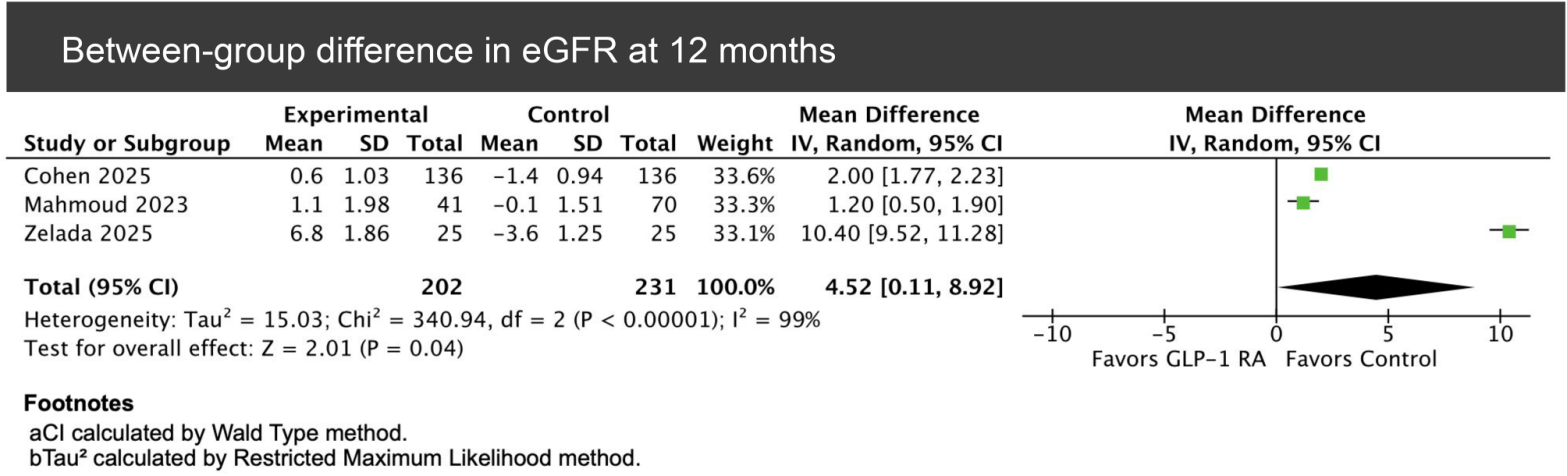
Forest plot comparing eGFR at 12 months between GLP-1RA users and matched control kidney transplant recipients (between-group).

In the comparative analysis between GLP-1RA users and matched control kidney-transplant recipients, pooled data from three studies [6, 15, 23] showed a significant trend toward higher eGFR among patients receiving GLP-1RAs (MD = 4.52; 95% CI 0.11–8.92, *P* = .04; Fig. 4).

**4. Within group estimated glomerular filtration rate at 3, 6, 12, and 24 months**

Because several studies did not include a comparator arm, we additionally summarized within-group changes in eGFR; these findings are descriptive and should be interpreted cautiously.

**Figure 5:**
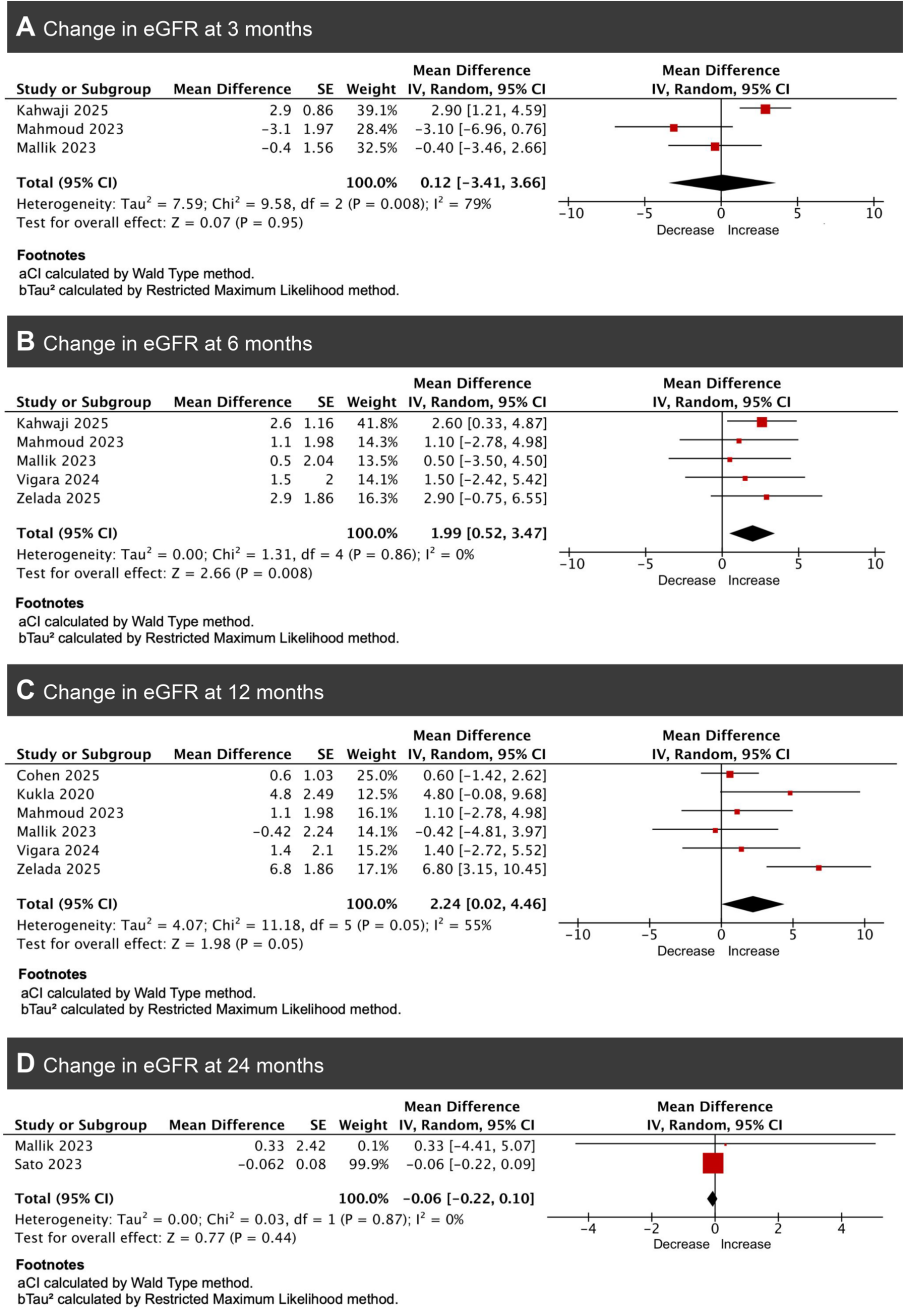
Forest plots of pooled mean change from baseline in eGFR among GLP-1RA-treated kidney transplant recipients (within-group) at (A) 3 months, (B) 6 months, (C) 12 months, and (D) 24 months after initiation of therapy.

From baseline to 3 months, pooled analysis of three studies showed no significant change in eGFR among kidney transplant recipients treated with GLP-1 receptor agonists [10, 15, 17]. The combined mean change in eGFR was +0.12 ml/min/1.73 m² (95% CI −3.41 to +3.66, *P* = .95). Between-study heterogeneity was high (*I*² = 79%, *P* = .008) (Fig. 5A). Since only three studies reported eGFR change at 3 months, formal sensitivity or subgroup analyses were not feasible. The high heterogeneity likely reflects genuine variation in study populations and measurement methods rather than bias.

From baseline to 6 months, pooled analysis of five studies demonstrated a significant improvement in eGFR among kidney transplant recipients treated with GLP-1 receptor agonists [10, 15, 17, 23, 27]. The combined mean increase in eGFR from baseline to 6 months was +1.99 ml/min/1.73 m² (95% CI 0.52–3.47, *P* = .008). Heterogeneity across studies was negligible (*I*² = 0%, *P* = .86) (Fig. 5B).

From baseline to 12 months, pooled analysis of six studies [6, 12, 15, 17, 22, 23] showed a significant improvement in eGFR among kidney transplant recipients receiving GLP-1 receptor agonists. The combined mean increase in eGFR from baseline to 12 months was +2.24 ml/min/1.73 m² (95% CI 0.02–4.46, *P* = .05). Substantial heterogeneity was observed (*I*² = 55%, *P* = .05) (Fig. 5C). Leave-one-out sensitivity analysis showed that excluding the study by Zelada *et al*. reduced heterogeneity from 55% to 0%, implying that this study was the primary source of between-study variability.

From baseline to 24 months, pooled analysis of two studies showed no significant change in eGFR among kidney transplant recipients treated with GLP-1 receptor agonists [17, 28]. The combined mean change in eGFR from baseline to 24 months was −0.06 ml/min/1.73 m² (95% CI −0.22 to 0.10, *P* = .44), indicating stable kidney function over long-term follow-up. There was no evidence of heterogeneity (*I*² = 0%, *P* = .87) (Fig. 5D).

**5. Graft outcomes**

GLP-1 RA use in kidney transplant recipients was consistently associated with preserved graft function and reduced graft loss. Four studies reported stable eGFR over time with GLP-1 RA therapy [10, 16, 17, 20] and no graft failures during follow-up periods ranging from 6 to 48 months. Observational data showed lower graft loss among GLP-1 RA users compared to non-users: Sahi *et al*. reported zero graft failures versus 5.7% in controls, and Orandi *et al*. found a 5-year cumulative incidence of death-censored graft loss of 7.5% versus 12.2%, with adjusted subhazard ratios of 0.55 (95% CI 0.40–0.76) and 0.51 (95% CI 0.36–0.71) after accounting for post-transplant insulin use. Additionally, a retrospective cohort study by Cohen *et al*. found that GLP-1 RA therapy was associated with a lower risk of a composite renal outcome, including graft rejection, dialysisn, retransplantation, or all-cause mortality, with a reported HR of 0.489 (95% CI 0.271–0.883) [6]. Overall, GLP-1 RA use appears to support graft preservation and reduce adverse renal outcomes across both short- and long-term follow-up.

**6. HbA1c percentage change**

**Figure 6:**
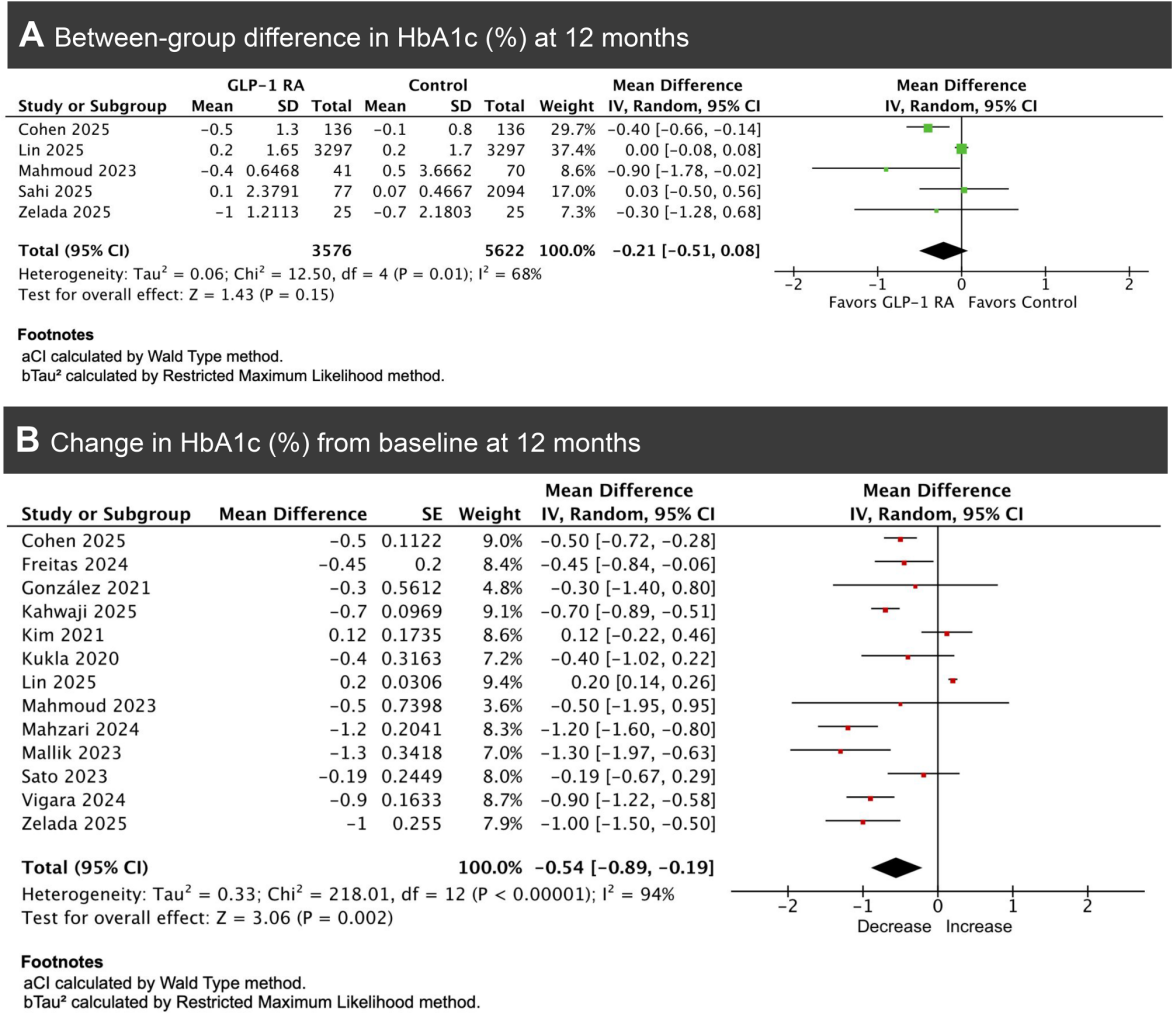
Forest plots of pooled HbA1c% change: (A) between-group comparison (GLP-1RA users vs. controls) and (B) within-group mean change from baseline among GLP-1RA users.

Pooled analysis of five studies demonstrated a non-significant reduction in HbA1c levels among GLP-1RA users compared with controls (MD = −0.21%, 95% CI −0.51 to 0.08, *P* = .15, *I*² = 68%; Fig. 6A) [6, 13, 15, 19, 23]. Leave-one-out analysis showed that exclusion of Lin *et al*. reduced heterogeneity to *I*² = 17% and altered the pooled estimate from −0.21 to −0.34, implying that the heterogeneity was largely driven by this study.

Within-group analysis of 13 studies demonstrated a significant reduction in HbA1c from baseline following 12 months of GLP-1RA treatment (MD = −0.54%, 95% CI −0.89 to −0.19, *P* = .002), with considerable heterogeneity (*I*² = 94%) (Fig. 6B) [6, 8–13, 15–17, 20, 22, 23, 29]. Leave-one-out sensitivity analysis identified Lin *et al*. as contributing most to heterogeneity; excluding this study reduced *I*² from 94% to 74% and shifted the pooled estimate from −0.54% (95% CI −0.89 to −0.19) to −0.62% (95% CI −0.85 to −0.39).

**7. Body mass index change**

**Figure 7:**
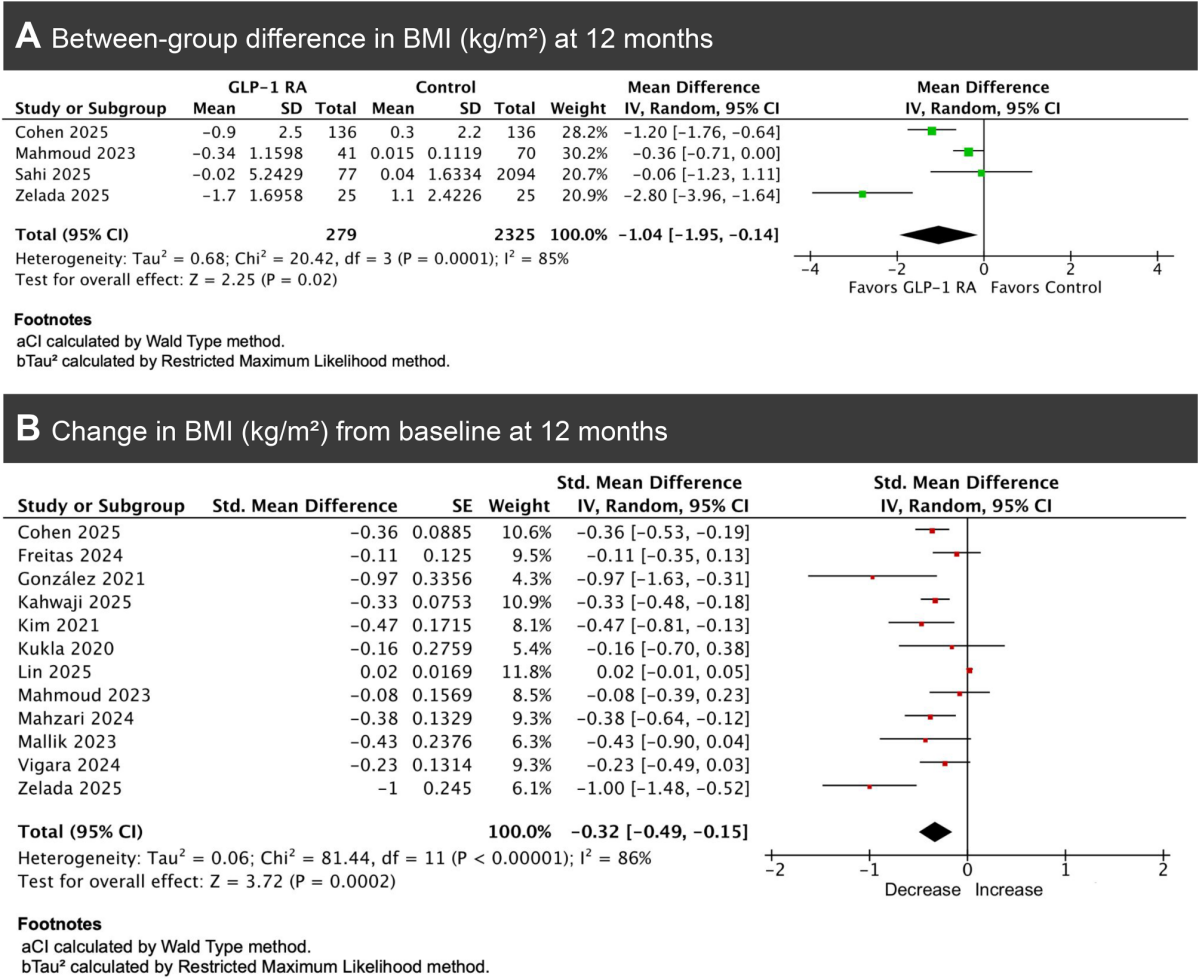
Forest plots showing pooled BMI change: (A) between-group comparison (GLP-1RA users vs. controls) and (B) within-group mean change from baseline among GLP-1RA users.

Pooled analysis of four studies demonstrated a significant reduction in BMI among GLP-1RA users compared with controls (MD = −1.04 kg/m², 95% CI −1.95 to −0.14, *P* = .02, *I*² = 85%; Fig. 7A) [6, 15, 19, 23]. Leave-one-out sensitivity analysis resulted in no significant change in heterogeneity.

Within-group meta-analysis, including 12 studies, showed a significant decrease in BMI from baseline after 12 months of GLP-1RA treatment (SMD = −0.32, 95% CI −0.49 to −0.15, *P* = .0002, Fig. 7B) [6, 8–12, 15, 17, 22, 23, 29, 30]. Leave-one-out sensitivity analysis identified Lin *et al*. as contributing most to heterogeneity; excluding it reduced *I*² from 86% to 47%, without materially altering the pooled estimate.

**8. Tacrolimus trough levels**

**Figure 8:**
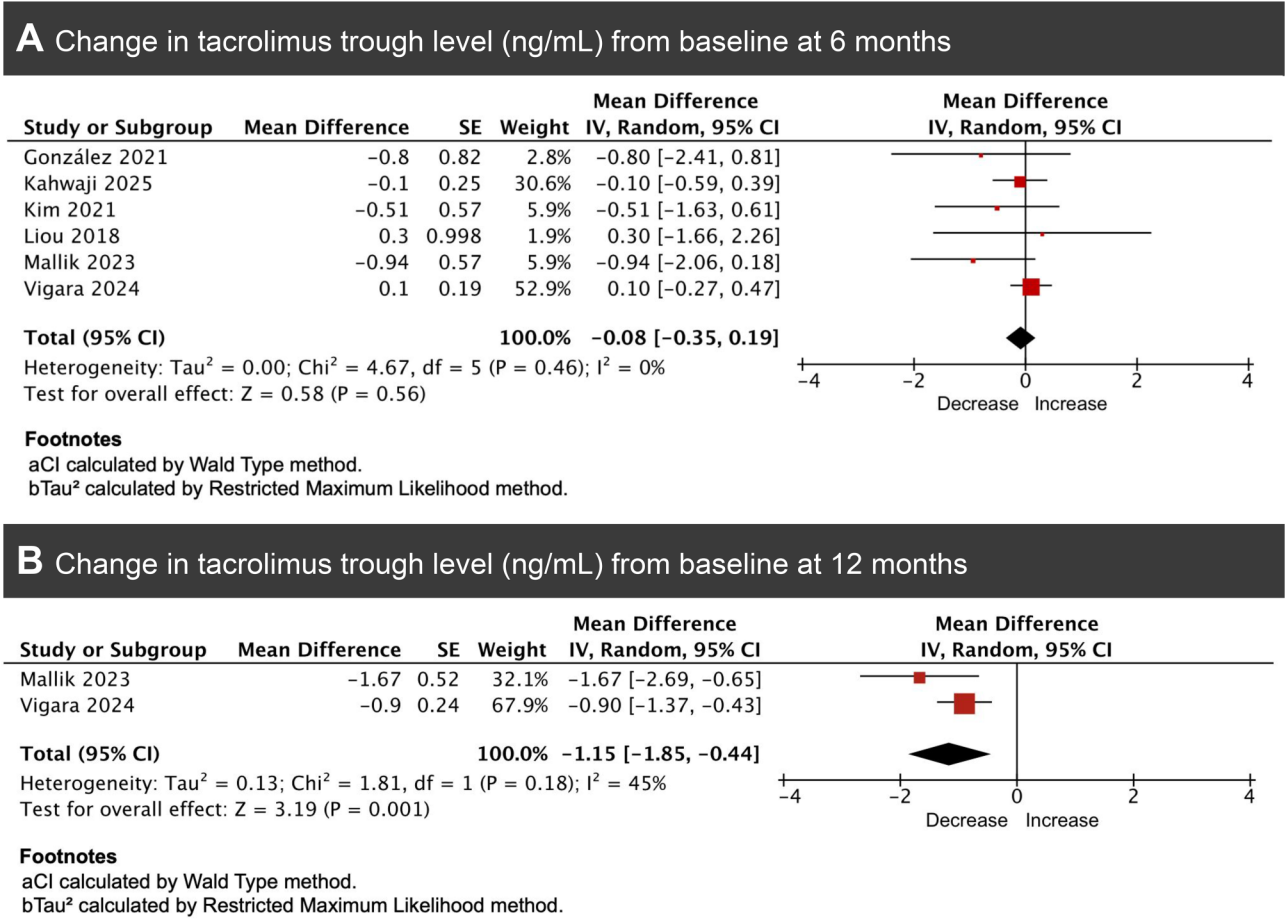
Forest plots of pooled mean change from baseline in tacrolimus trough levels among kidney transplant recipients treated with GLP-1RA therapy at (A) 6 months and (B) 12 months of follow-up.

At 6 months, six studies reported changes in tacrolimus trough levels among kidney transplant recipients treated with GLP-1 receptor agonists [6, 10, 14, 17, 21, 22]. The pooled mean difference was −0.08 ng/ml (95% CI −0.35 to 0.19, *P* = .56). Heterogeneity was negligible (*I*² = 0%) (Fig. 8A). Subgroup analyses confirmed consistently low heterogeneity across follow-up categories.

At 1 year, two studies provided follow-up data, showing a pooled mean difference of −1.15 ng/ml (95% CI −1.85 to −0.44, *P* = .001), suggesting a modest but statistically significant decrease in tacrolimus levels over longer-term follow-up, with no substantial heterogeneity (*I*² = 45%) (Fig. 8B) [17, 21].

**9. Total daily insulin dose**

**Figure 9:**
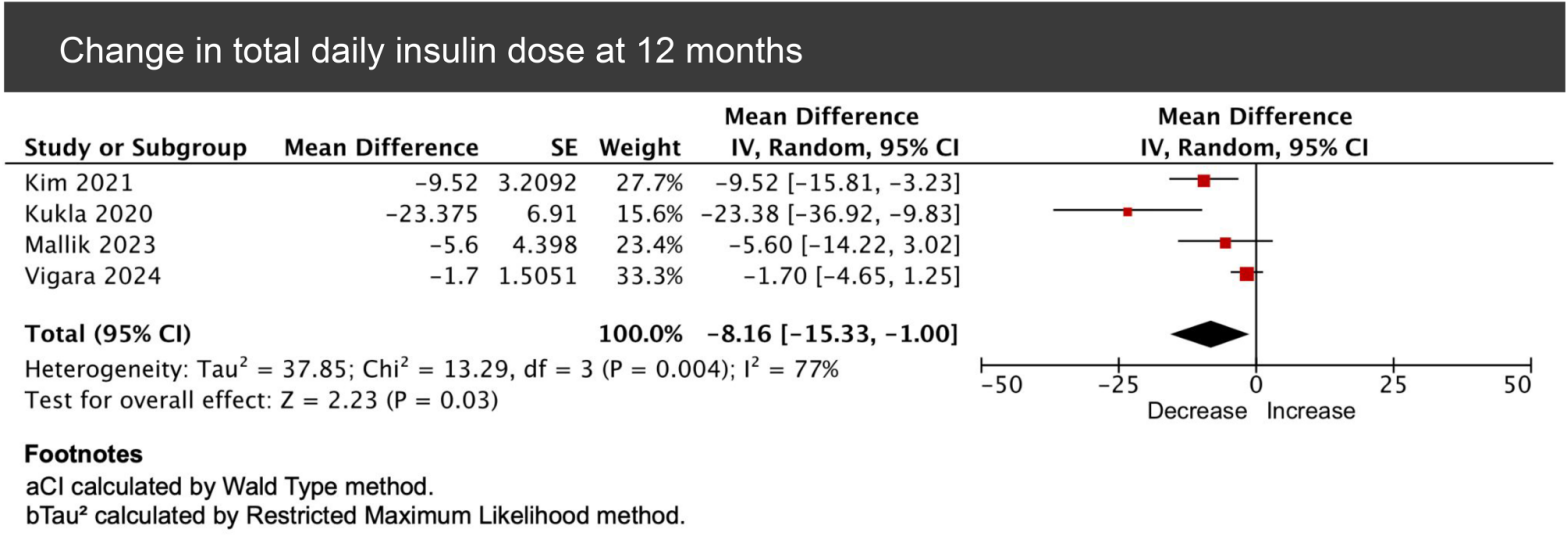
Forest plot of pooled mean change from baseline in total daily insulin dose among GLP-1RA-treated kidney transplant recipients at 12 months.

Within-group analysis of four studies reported a pooled mean difference of -8.16 units of insulin (95% CI -15.33 to -1.00, *P* = .03) after 1 year of follow-up. Heterogeneity was substantial (*I*² = 77%) (Fig. 9). Leave-one-out sensitivity analysis did decrease heterogeneity, but it remained substantial, suggesting that variability was not driven by a single study but rather by differences in study design and patient characteristics.

**10. Urinary albumin–creatinine ratio**

**Figure 10:**
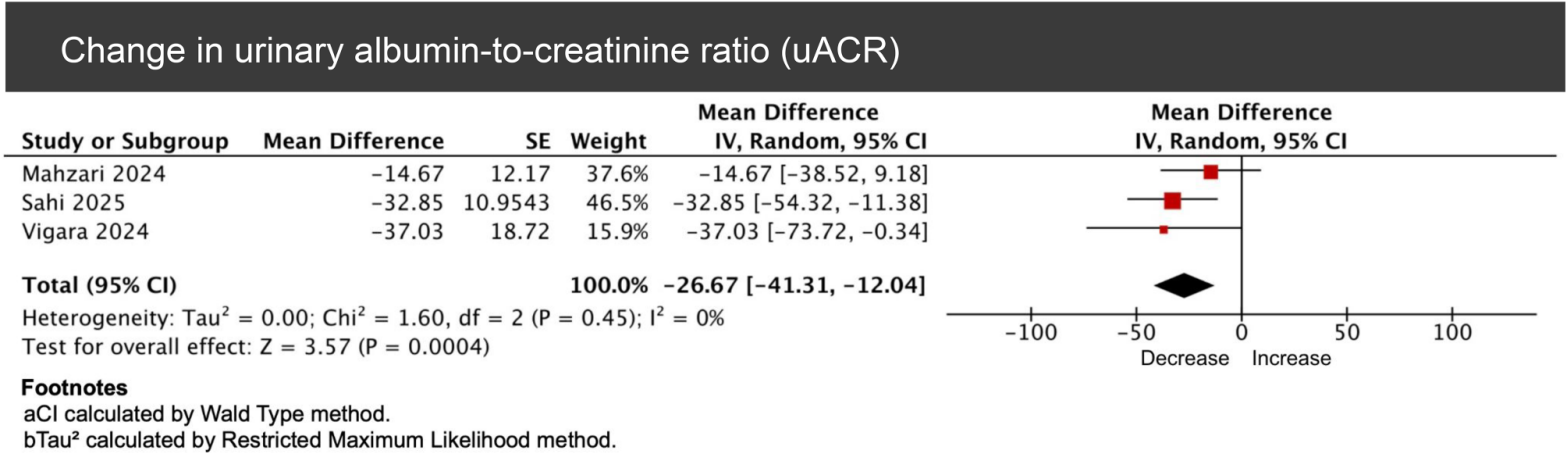
Forest plot of pooled mean change from baseline in uACR among kidney transplant recipients treated with GLP-1 receptor agonists.

Three studies reported the mean difference in urinary albumin–creatinine ratio (uACR), with a pooled value of -26.67 (95% CI -41.31 to -12.04, *P* = .0004, *I*² = 0%; Fig. 10) [16, 19, 21]. Furthermore, Liou *et al*. reported no significant change in urinary protein–creatinine ratio (uPCR) during a mean follow-up period was 19.4 ± 7.6 [14]. Mallik *et al*. also reported an insignificant MD of uPCR in mg/mmol of 12.73 ± 42.82 (*P* = .21) at 1 year and of 1.2 ± 21.4 (*P* = .87) at 2 years [17]. Conversely, Zelada *et al*. reported a significant change in uPCR 12 months after starting GLP1-RA therapy, with a mean decrease of 128.4 in the GLP1-RA group and an average increase of 15.4 mg/dl in the control group (*P* < .01) [23].

**11. Gastrointestinal side effects or complications**

Nine studies reported gastrointestinal (GI) adverse events [10–12, 14–16, 19, 22, 23]. Across studies, GI side effects were the most frequently observed adverse events associated with GLP-1 receptor agonists in kidney transplant recipients and were generally mild and self-limited. Reported symptoms included nausea, vomiting, diarrhea, and reduced appetite.

The incidence of GI intolerance ranged from ∼10% to 20%. Most cases were transient or improved with dose reduction. Discontinuation due to GI symptoms was uncommon in most cohorts; however, higher discontinuation rates were observed in isolated studies, primarily driven by persistent intolerance. No study reported GI-related hospitalizations or severe complications. A detailed report of gastrointestinal side effects can be found in Supplementary Table S7.

**12. Pancreatic complications**

Six studies reported data on pancreatic complications [6, 12, 13, 18, 22]. In Orandi *et al*., pancreatitis occurred at a rate of 8.8 per 1000 person-years among GLP-1RA users versus 8.2 per 1000 in non-users. The initially observed higher risk (aHR 1.82) became non-significant after insulin adjustment (aHR 1.29; *P* = .32) [18]. In Cohen *et al*., biliopancreatic events, including pancreatitis, occurred less frequently in GLP-1RA users compared with matched non-user kidney transplant recipients (0.8 vs. 3.6 events per 100 patient-years, *P* < .001) [6]. Lin *et al*. reported no increased risk of pancreatitis compared to controls [13]. In Kukla *et al*., one kidney transplant recipient (6%) developed clinical acute pancreatitis while receiving liraglutide 1.8 mg daily, which was resolved after discontinuation of the drug [12]. In the two Vigara *et al*. prospective cohort studies, no cases of pancreatitis were observed during follow-up. One patient developed pancreatic adenocarcinoma after 8 months on dulaglutide, but this event was not attributed to GLP-1RA therapy [21, 22].

**13. Urogenital infections**

Four studies reported data on urogenital infections [6, 9, 15, 17]. In the study by Mahmoud *et al*., among 41 kidney transplant recipients treated with GLP-1 receptor agonists, urinary tract infections occurred in 2 patients (4.9%); in comparison, 3 of 70 patients (4.3%) in the control group experienced urinary tract infections, with no genital infections observed in either group [15]. In the Cohen *et al*. matched-cohort study (*n* = 136 GLP-1RA users vs. 136 controls), the risk of the composite outcome of genitourinary infection or all-cause mortality was not elevated [6]. In the Mallik *et al*. UK cohort of 23 renal transplant recipients receiving GLP-1 receptor agonists, urinary tract infections were observed in two patients (8.7%) during follow-up [17]. Gonzales *et al*. reported a case of discontinuation of the drug after developing urinary sepsis requiring hospital admission [31].

**14. Opportunistic infections**

Two studies reported data on opportunistic infections [17, 23]. Zelada *et al*., in a single-center analysis, reported no difference in rates of BK-virus or cytomegalovirus infections reported in GLP-1RA users and non-users [23]. In Mallik *et al*., no cases of cytomegalovirus infection or BK viraemia were reported throughout the study period [17].

**15. Discontinuation of GLP-1RA**

Eleven studies reported data on discontinuation [8, 9, 11–13, 15–17, 19, 22, 23]. Freitas *et al*., Zelada *et al*., and Mahmoud *et al*. reported no discontinuations [8, 15, 23]. Sahi *et al*. reported 6% of patients stopping treatment for side effects and 3% for insurance reasons [19]. Kim *et al*. and Mahzari *et al*. each described small proportions (3%–5%) discontinuing for GI intolerance or cost [11, 16]. Kukla *et al*. and Vigara *et al*. reported higher discontinuation rates (29% and 11 of 16 patients, respectively), mainly due to GI symptoms [12, 22]. Other studies, including Lin *et al*., Mallik *et al*., and Gonzalez *et al*., provided limited data or described isolated discontinuations unrelated to drug toxicity [9, 13, 17]. Overall, treatment discontinuation was generally uncommon and primarily attributed to GI intolerance or financial issues.

**16. Hypoglycemia**

Six studies reported data on hypoglycemia [11, 24, 27, 31–33]. Lin *et al*. reported no increased risk of hypoglycemia between GLP-1RA users compared to controls [13]. Liou *et al*. and Gonzales *et al*. reported no episodes of hypoglycemia [9, 14]. In Kukla *et al*., 6 of 14 patients (43%) experienced mild-to-moderate hypoglycemia (1–3 times per week) before GLP-1RA initiation; after 12 months, 4 had complete resolution and 2 showed improvement (reduced to 1–2 times per month), with a concurrent median insulin dose reduction of 30 IU (*P* = .007) [12]. In Kim *et al*., mild hypoglycemia occurred in three patients during the 6-month follow-up, while no episodes of severe hypoglycemia requiring hospitalization were observed [11]. In Vigara *et al*., one patient discontinued GLP-1RA therapy due to hypoglycemia; however, no severe or recurrent hypoglycemic episodes were reported in the cohort [22].

### Findings from conference abstracts and posters

Several abstracts and conference posters on GLP-1RA use in kidney transplant recipients were identified (Supplementary Table S8). Although not eligible for quantitative pooling, these materials consistently reported modest weight loss, improved glycemic control, and generally stable kidney function over short follow-up. A few abstracts noted reductions in proteinuria, and no clear signals of increased rejection, de novo donor-specific antibody formation, or calcineurin-inhibitor instability were observed. Safety findings were limited to mild gastrointestinal intolerance, with rare, isolated events. Cardiovascular and hospitalization outcomes were sparsely reported and descriptive. Given their small sample sizes and limited methodological detail, these abstracts were summarized narratively but not incorporated into the main analyses.

### Certainty of evidence

Based on the GRADE evaluation, the certainty of evidence ranged from moderate to very low across outcomes. Certainty was generally downgraded for risk of bias, inconsistency, and imprecision related to retrospective study design. Mortality and cardiovascular outcomes reached moderate certainty due to consistent direction and magnitude of benefit across large cohorts. In contrast, metabolic outcomes such as HbA1c, BMI, and insulin requirement were rated low because of high heterogeneity and imprecision. Renal and graft outcomes were rated low to very low because of smaller sample sizes and variation in follow-up. Detailed GRADE ratings are shown in Supplementary Table S5.

### Sensitivity analyses

Sensitivity analyses using fixed-effect models yielded estimates in the same direction as the primary random-effects (REML) analyses, with mostly no changes in statistical significance. Results are summarized in Supplementary Table S9.

### Assessment of publication bias

Funnel plots for the primary and secondary outcomes appeared broadly symmetrical (Supplementary Fig. S1A and B). Egger’s regression tests were conducted only for outcomes with ≥10 studies. For the BMI change within-group analysis, the intercept was –2.75 (*P* = .0004; *R*² = 0.31), and for the HbA1c change within-group analysis, the intercept was –3.98 (*P* = .005; *R*² = 0.32), both indicating possible small-study effects.

## DISCUSSION

This systematic review and meta-analysis represent the most comprehensive synthesis to date evaluating the safety, efficacy, and clinical outcomes of GLP-1RA therapy in kidney transplant recipients. While the cardiovascular and renal benefits of GLP-1RAs are well established in the general population, evidence among kidney transplant recipients has remained fragmented and inconclusive due to small sample sizes, retrospective designs, and heterogeneous outcomes. The current analysis addresses this critical knowledge gap by integrating data from 17 studies encompassing 54 680 kidney transplant recipients, providing quantitative estimates across metabolic, renal, cardiovascular, and safety endpoints.

Our pooled data demonstrate that GLP-1RA use in kidney transplant recipients is associated with a significant reduction in all-cause mortality and a reduced risk of MACE. These findings mirror the robust cardioprotective effects established in large cardiovascular outcome trials among patients with type 2 diabetes [34–36]. The cardiovascular benefits in kidney transplant recipients likely arise from a combination of improved metabolic control [37, 38], attenuation of systemic inflammation [39], and favorable hemodynamic effects on both cardiac [40, 41] and renal microcirculation [42]. Mechanistically, GLP-1RAs enhance capillary perfusion [43], reduce oxidative stress [44], and inhibit vascular smooth muscle proliferation [45], factors known to mitigate post-transplant cardiovascular burden, which remains a leading cause of mortality in this population.

Serial analyses revealed that eGFR remained stable at 3 months, improved modestly at 6 and 12 months (+1.99 and +2.24 ml/min/1.73 m², respectively), and remained preserved at 24 months, suggesting that GLP-1RAs neither impair graft function nor induce nephrotoxicity. The effect of GLP-1RAs on eGFR alongside metabolic, immunosuppressive and safety effects are summarized in Supplementary Table S6. The stabilization of eGFR may stem from renoprotective effects regardless of metabolic status [46], reduced glomerular pyroptosis [47], and attenuation of tubulointerstitial inflammation [48, 49] mediated through GLP-1RA-induced natriuresis [50] and suppression of the renin–angiotensin–aldosterone system. Moreover, GLP-1RA therapy was consistently associated with lower graft loss and reduced composite renal outcomes such as graft rejection or dialysis re-initiation, indicating potential graft-protective properties. These findings are biologically plausible given that GLP-1R signaling in renal tissue modulates oxidative stress [51] and cytokine production [52], thereby protecting tubular epithelial cells against ischemia-reperfusion [53] and renal fibrosis [54]. Additionally, modest reductions in uACR suggest early nephroprotective effects mediated through reduced glomerular hyperfiltration [55] and anti-inflammatory actions.

GLP-1 receptor agonist therapy produced a significant improvement in glycemic control, with a pooled mean HbA1c reduction of −0.51% from baseline and consistent trends favoring GLP-1RA users in comparative analyses (Supplementary Table S6). This effect is particularly valuable in kidney transplant recipients, where glycemic regulation is challenged by corticosteroid- and calcineurin inhibitor-induced insulin resistance. GLP-1RAs enhance glucose-dependent insulin secretion, suppress inappropriate glucagon release, and slow gastric emptying, thereby reducing both fasting and postprandial glucose levels [56, 57]. The observed reduction in daily insulin requirements reflects an insulin-sparing effect that helps limit insulin resistance [58, 59] and glycemic variability [60], both important contributors to graft and cardiovascular complications. In parallel, GLP-1RAs achieved a significant decrease in BMI consistent with their established weight-lowering effects in non-transplant populations [61]. Weight loss is mediated centrally via hypothalamic GLP-1 receptor activation related to satiety, which inhibits orexigenic and stimulates anorexigenic neurons, and reduces caloric intake [62, 63]. This mechanism remains effective despite steroid use or metabolic derangements after transplantation. Improved weight control subsequently enhances blood pressure [64] and lipid profile [65], contributing to lower cardiometabolic risk.

An essential clinical concern regarding GLP-1RA use in kidney transplant recipients is the potential interaction with tacrolimus, the mainstay of immunosuppressive therapy. Our pooled results showed no significant change in tacrolimus trough levels at 6 months and only a modest decrease at 1 year, which was not associated with graft dysfunction or rejection. The likely mechanism is delayed gastric emptying that can transiently affect tacrolimus absorption without altering overall exposure. These data suggest that GLP-1RAs can be co-administered safely with close therapeutic drug monitoring during initiation (Supplementary Table S6).

Our findings are consistent with the prior systematic reviews and meta-analyses evaluating incretin-based therapies in kidney transplant recipients. The Clinical Kidney Journal 2024 meta-analysis reported improvements in HbA1c and weight and a reduction in proteinuria without a clear signal of tacrolimus instability [7]. More recently, Lee *et al*. evaluated both SGLT2 inhibitors and GLP-1 receptor agonists and similarly demonstrated favorable cardiometabolic and renal outcomes in transplant recipients [66]. Compared with these earlier syntheses (search through May 2023 and February 2025, respectively), our analysis incorporates a broader and more recent evidence base, including larger cohorts and additional transplant-specific endpoints [6, 66]. By focusing exclusively on GLP-1RA therapy, our analysis provides a more granular evaluation of graft function trajectories, immunosuppressive parameters, metabolic outcomes, and safety. This expanded dataset also enabled a more detailed GRADE assessment of outcome certainty while still acknowledging the observational nature and residual confounding of the available literature.

### Strengths and limitations

The primary strength of this meta-analysis lies in its comprehensive inclusion of diverse outcome domains, metabolic, renal, cardiovascular, and safety, providing a holistic understanding of GLP-1RA therapy in kidney transplant recipients. Because the transplant literature remains sparse and outcomes are reported inconsistently, we did not designate a single primary endpoint; instead, we synthesized clinically relevant outcome domains and interpret endpoints supported by few studies as exploratory.

As such, several limitations should be acknowledged when interpreting the results of this meta-analysis. Most included studies were retrospective and observational, introducing potential selection bias, confounding by indication, and unmeasured covariates that could influence treatment assignment and outcomes. There was substantial heterogeneity in study design, including variability in sample size, follow-up duration, and baseline characteristics such as kidney function, diabetes status, and immunosuppressive regimens. The studies also differed in the type, dose, and timing of GLP-1RA initiation after transplantation, which may have contributed to between-study variability. In addition, most reports relied on short- to medium-term outcomes, limiting the ability to assess long-term effects on graft survival and cardiovascular events. Some analyses were underpowered for infrequent adverse events, such as pancreatitis or opportunistic infections, reducing the precision of pooled estimates. The use of aggregate data rather than individual patient data precluded more granular subgroup analyses, including evaluation by sex, donor type, or concurrent use of other antidiabetic agents. Although we explored heterogeneity by stratifying certain outcomes by follow-up time, more detailed subgroup analyses and meta-regression were not feasible or reliable because potential effect modifiers were inconsistently reported across studies and many endpoints included few studies, limiting statistical power and increasing the risk of misleading associations.

Many included studies were single-arm or did not report sufficient comparator data, so several outcomes could only be summarized using within-group pre–post changes. Such analyses are vulnerable to regression to the mean, secular trends, changes in concomitant therapy, and selection/survivor bias, and therefore should be interpreted as descriptive rather than causal. Several endpoints were informed by few studies, and small-*k* random-effects inference can be imprecise; sensitivity analyses comparing fixed-effect and random-effects models were performed, but residual uncertainty remains and findings should be considered exploratory. Furthermore, publication bias and potential cohort overlap in multicenter registry-based studies cannot be completely excluded. Finally, differences in laboratory methods, endpoint definitions, and reporting standards may have influenced effect estimates. These limitations highlight the need for prospective, randomized, and adequately powered trials with standardized endpoints to confirm the safety and efficacy of GLP-1 receptor agonists in kidney transplant recipients.

## CONCLUSION

In summary, the available evidence suggests that GLP-1 receptor agonist use in kidney transplant recipients is associated with improvements in glycemic control and weight and does not show a consistent signal of adverse effects on graft function or immunosuppressive stability in the short to medium term. Evidence for hard clinical outcomes remains limited and should be interpreted cautiously. GLP-1RAs may be considered as part of individualized post-transplant metabolic management, with close clinical monitoring during initiation and dose escalation. Larger prospective comparative studies and randomized trials are needed to better define long-term safety and efficacy in this population.

## Supplementary Material

sfag105_Supplemental_File

## Data Availability

The authors confirm that the data supporting the findings of this study are available within the article and its Supplementary material.

## Notes

PROSPERO registration number: CRD420251153352

## References

[1] Kanbay M, Siriopol D, Guldan M et al. Prognostic impact of post-transplant diabetes mellitus in kidney allograft recipients: a meta-analysis. Nephrol Dial Transplant. 2025;40(3):554–76. 10.1093/ndt/gfae185. 39134508 PMC11879034

[2] Kanbay M, Siriopol D, Mahmoud A-RS et al. Impact of weight change on kidney transplantation outcomes: a systematic review and meta-analysis. Diabetes Obes Metab. 2025;27:1369–78. 10.1111/dom.16135. 39691978

[3] Giugliano D, Maiorino MI, Bellastella G et al. GLP-1 receptor agonists for prevention of cardiorenal outcomes in type 2 diabetes: an updated meta-analysis including the REWIND and PIONEER 6 trials. Diabetes Obes Metab. 2019;21:2576–80. 10.1111/dom.13847. 31373167

[4] Ertuglu LA, Porrini E, Hornum M et al. Glucagon-like peptide-1 receptor agonists and sodium-glucose cotransporter 2 inhibitors for diabetes after solid organ transplantation. Transpl Int. 2021;34:1341–59. 10.1111/tri.13883. 33880815

[5] Mahzari MM, Alluhayyan OB, Almutairi MH et al. Safety and efficacy of semaglutide in post kidney transplants patients with type 2 diabetes or post-transplant diabetes. J Clin Transl Endocrinol. 2024;36:100343. 10.1016/j.jcte.2024.100343. 38623181 PMC11016780

[6] Diker CT, Rudman Y, Turjeman A et al. Glucagon-like peptide 1 receptor agonists and renal outcomes in kidney transplant recipients with diabetes mellitus. Diabetes Metab. 2025;51:101624. 10.1016/j.diabet.2025.101624. 39961479

[7] Krisanapan P, Suppadungsuk S, Sanpawithayakul K et al. Safety and efficacy of glucagon-like peptide-1 receptor agonists among kidney transplant recipients: a systematic review and meta-analysis. Clin Kidney J. 2024;17:sfae018. 10.1093/ckj/sfae018. 38410684 PMC10896177

[8] Freitas J, Silvano J, Ribeiro C et al. Glucagon-like peptide-1 receptor agonists in kidney transplant recipients—a retrospective single center study. Braz J Transplant. 2024;27:e1224.

[9] Yugueros GA, Kanter J, Sancho A et al. Institutional experience with new antidiabetic drugs in kidney transplant. Transplant Proc. 2021;53:2678–80. 10.1016/j.transproceed.2021.08.042. 34615601

[10] Kahwaji J, Hashmi S, Parke CY et al. Safety and efficacy of liraglutide and semaglutide in kidney transplant recipients. Kidney360. 2025;6:848–50. 10.34067/KID.0000000706. 39847451 PMC12136651

[11] Kim HS, Lee J, Jung CH et al. Dulaglutide as an effective replacement for prandial insulin in kidney transplant recipients with type 2 diabetes mellitus: a retrospective review. Diabetes Metab J. 2021;45:948–53. 10.4093/dmj.2020.0180. 33535737 PMC8640157

[12] Kukla A, Hill J, Merzkani M et al. The use of GLP1R agonists for the treatment of type 2 diabetes in kidney transplant recipients. Transplant Direct. 2020;6:e524. 10.1097/TXD.0000000000000971. 32095510 PMC7004635

[13] Lin LC, Chen JY, Huang TT et al. Association of glucagon-like peptide-1 receptor agonists with cardiovascular and kidney outcomes in type 2 diabetic kidney transplant recipients. Cardiovasc Diabetol. 2025;24:87. 10.1186/s12933-025-02649-0. 39984953 PMC11846168

[14] Liou JH, Liu YM, Chen CH. Management of diabetes mellitus with glucagonlike peptide-1 agonist liraglutide in renal transplant recipients: a retrospective study. Transplant Proc. 2018;50:2502–5. 10.1016/j.transproceed.2018.03.087. 30316386

[15] Mahmoud T, Yagan J, Hasan A et al. Sodium-glucose co-transporter 2 inhibitors and glucagon-like peptide-1 receptor agonists, efficacy and safety in diabetic kidney transplant recipients. Clin Transplant. 2023;37:e15144. 10.1111/ctr.15144. 37755118

[16] Mahzari MM, Alluhayyan OB, Almutairi MH et al. Safety and efficacy of semaglutide in post kidney transplant patients with type 2 diabetes or post-transplant diabetes. J Clin Transl Endocrinol. 2024;36:100343. 38623181 10.1016/j.jcte.2024.100343 PMC11016780

[17] Mallik R, Ali O, Casabar M et al. Glucagon-like peptide-1 receptor analogues in renal transplant recipients with diabetes: medium term follow of patients from a single UK centre. Diabet Med. 2023;40:e15057. 10.1111/dme.15057. 36721974

[18] Orandi BJ, Chen Y, Li Y et al. GLP-1 receptor agonists in kidney transplant recipients with pre-existing diabetes: a retrospective cohort study. Lancet Diabetes Endocrinol. 2025;13:374–83. 10.1016/S2213-8587(24)00371-1. 40056927 PMC12171561

[19] Sahi SS, Garcia VO, Na J et al. Benefits of glucagon-like peptide-1 receptor agonists after kidney transplantation. Endocr Pract. 2025;31:798–804. 10.1016/j.eprac.2025.02.020. 40054529

[20] Sato T, Azuma Y, Ozone C et al. Possible advantage of glucagon-like peptide 1 receptor agonists for kidney transplant recipients with type 2 diabetes. J Clin Endocrinol Metab. 2023;108:2597–603. 10.1210/clinem/dgad177. 36974363

[21] Vigara LA, Villanego F, Orellana C et al. Effectiveness and safety of glucagon-like peptide-1 receptor agonist in a cohort of kidney transplant recipients. Clin Transplant. 2022;36:e14633. 10.1111/ctr.14633. 35258121

[22] Vigara LA, Villanego F, Orellana C et al. Use of glucagon-like peptide type 1 receptor agonists in kidney transplant recipients. Nefrologia (Engl Ed). 2024;44:885–93. 10.1016/j.nefroe.2024.11.007. 39645509

[23] Zelada H, Campana M, Kawai K et al. Efficacy, tolerability, and safety of glucagon-like peptide 1 receptor agonists (GLP1-RA) in kidney transplant recipients with diabetes. Clin Transplant. 2025;39:e70144. 10.1111/ctr.70144. 40230336

[24] Lin LC, Chen JY, Huang TTM et al. Association of glucagon-like peptide-1 receptor agonists with cardiovascular and kidney outcomes in type 2 diabetic kidney transplant recipients. Cardiovasc Diabetol. 2025;24:87. 10.1186/s12933-025-02649-0 39984953 PMC11846168

[25] Orandi BJ, Chen YS, Li YT et al. GLP-1 receptor agonists in kidney transplant recipients with pre-existing diabetes: a retrospective cohort study. Lancet Diabetes Endocrinol. 2025;13:374–83. 10.1016/S2213-8587(24)00371-1. 40056927 PMC12171561

[26] Sahi SS, Valencia OG, Na J et al. Benefits of glucagon-like peptide-1 receptor agonists after kidney transplantation. Endocr Pract. 2025;31:798–804. 10.1016/j.eprac.2025.02.020. 40054529

[27] Vigara LA, Villanego F, Orellana C et al. Use of glucagon-like peptide type 1 receptor agonists in kidney transplant recipients. Nefrologia. 2024;44:885–93. 10.1016/j.nefro.2023.06.010. 39645509

[28] Sato T, Azuma Y, Ozone C et al. Possible advantage of glucagon-like peptide 1 receptor agonists for kidney transplant recipients with type 2 diabetes. J Clin Endocrinol Metab. 2023;108:2597–603. 10.1210/clinem/dgad177. 36974363

[29] Strom Halden TA, Asberg A, Vik K et al. Short-term efficacy and safety of sitagliptin treatment in long-term stable renal recipients with new-onset diabetes after transplantation. Nephrol Dial Transplant. 2014;29:926–33. 10.1093/ndt/gft536. 24452849

[30] Mohammed MM, Buraykan AO, Hamad AM et al. Corrigendum to “Safety and efficacy of semaglutide in post kidney transplants patients with type 2 diabetes or post-transplant diabetes [J. Clin. Translat. Endoc. 36C (2024) 100343]”. J. Clin. Transl. Endocrinol. 2024; 38:100360. 39764278 10.1016/j.jcte.2024.100360 PMC11701984

[31] González AY, Kanter J, Sancho A et al. Institutional experience with new antidiabetic drugs in kidney transplant. Transplant Proc. 2021;53:2678–80. 10.1016/j.transproceed.2021.08.042. 34615601

[32] Kukla A, Hill J, Merzkani M et al. The use of GLP1R agonists for the treatment of type 2 diabetes in kidney transplant recipients. Transplant Direct. 2020;6:e524. 32095510 10.1097/TXD.0000000000000971 PMC7004635

[33] Liou JH, Liu YM, Chen CH. Management of diabetes mellitus with glucagonlike peptide-1 agonist liraglutide in renal transplant recipients: a retrospective study. Transplant Proc. 2018;50:2502–5. 10.1016/j.transproceed.2018.03.087. 30316386

[34] Verma S, Bain SC, Buse JB et al. Occurrence of first and recurrent major adverse cardiovascular events with liraglutide treatment among patients with type 2 diabetes and high risk of cardiovascular events: a post hoc analysis of a randomized clinical trial. JAMA Cardiol. 2019;4:1214–20. 10.1001/jamacardio.2019.3080. 31721979 PMC6865601

[35] Green JB, Everett BM, Ghosh A et al. Cardiovascular outcomes in GRADE (Glycemia Reduction Approaches in Type 2 Diabetes: A Comparative Effectiveness Study). Circulation. 2024;149:993–1003. 10.1161/CIRCULATIONAHA.123.066604. 38344820 PMC10978227

[36] Mentz RJ, Bethel MA, Merrill P et al. Effect of once-weekly exenatide on clinical outcomes according to baseline risk in patients with type 2 diabetes mellitus: insights from the EXSCEL trial. J Am Heart Assoc. 2018;7:e009304. 10.1161/JAHA.118.009304. 30371301 PMC6404902

[37] Abasheva D, Ortiz A, Fernandez-Fernandez B. GLP-1 receptor agonists in patients with chronic kidney disease and either overweight or obesity. Clin Kidney J. 2024;17:19–35. 10.1093/ckj/sfae296. 39583142 PMC11581768

[38] Gajjar A, Raju AK, Gajjar A et al. SGLT2 inhibitors and GLP-1 receptor agonists in cardiovascular-kidney-metabolic syndrome. Biomedicines. 2025;13:1924. 10.3390/biomedicines13081924 40868177 PMC12383900

[39] Ren Y, Chen Y, Zheng W et al. The effect of GLP-1 receptor agonists on circulating inflammatory markers in type 2 diabetes patients: a systematic review and meta-analysis. Diabetes Obes Metab. 2025;27:3607–26. 10.1111/dom.16366. 40230207

[40] Park B, Bakbak E, Teoh H et al. GLP-1 receptor agonists and atherosclerosis protection: the vascular endothelium takes center stage. Am J Physiol Heart Circ Physiol. 2024;326:H1159–h76. 10.1152/ajpheart.00574.2023. 38426865

[41] Król M, Kupnicka P, Żychowska J et al. Molecular insights into the potential cardiometabolic effects of GLP-1 receptor analogs and DPP-4 inhibitors. Int J Mol Sci. 2025;26:6777. 40725024 10.3390/ijms26146777 PMC12295648

[42] Hviid AVR, Sørensen CM. Glucagon-like peptide-1 receptors in the kidney: impact on renal autoregulation. Am J Physiol Renal Physiol. 2020;318:F443–f54. 10.1152/ajprenal.00280.2019. 31841385

[43] Smits MM, Muskiet MH, Tonneijck L et al. GLP-1 receptor agonist exenatide increases capillary perfusion independent of nitric oxide in healthy overweight men. Arterioscler Thromb Vasc Biol. 2015;35:1538–43. 10.1161/ATVBAHA.115.305447. 25908765

[44] Bray JJH, Foster-Davies H, Salem A et al. Glucagon-like peptide-1 receptor agonists improve biomarkers of inflammation and oxidative stress: a systematic review and meta-analysis of randomised controlled trials. Diabetes Obes Metab. 2021;23:1806–22. 10.1111/dom.14399. 33830637

[45] Lee J, Hong SW, Kim MJ et al. Glucagon-like peptide receptor agonist inhibits angiotensin II-induced proliferation and migration in vascular smooth muscle cells and ameliorates phosphate-induced vascular smooth muscle cells calcification. Diabetes Metab J. 2024;48:83–96. 10.4093/dmj.2022.0363. 38173373 PMC10850275

[46] Mendonça L, Moura H, Chaves PC et al. The impact of glucagon-like peptide-1 receptor agonists on kidney outcomes: a meta-analysis of randomized placebo-controlled trials. Clin J Am Soc Nephrol. 2024;20:159–68. 10.2215/CJN.0000000584. 39480988 PMC11835157

[47] Wu W, Wang Y, Shao X et al. GLP-1RA improves diabetic renal injury by alleviating glomerular endothelial cells pyrotosis via RXRα/circ8411/miR-23a-5p/ABCA1 pathway. PLoS One. 2024;19:e0314628. 10.1371/journal.pone.0314628. 39621727 PMC11611192

[48] Li R, She D, Ye Z et al. Glucagon-like peptide 1 receptor agonist improves renal tubular damage in mice with diabetic kidney disease. Diabetes Metab Syndr Obes. 2022;15:1331–45. 10.2147/DMSO.S353717. 35519661 PMC9064072

[49] Sourris KC, Ding Y, Maxwell SS et al. Glucagon-like peptide-1 receptor signaling modifies the extent of diabetic kidney disease through dampening the receptor for advanced glycation end products-induced inflammation. Kidney Int. 2024;105:132–49. 10.1016/j.kint.2023.09.029. 38069998

[50] Wajdlich M, Nowicki M. The impact of GLP-1 receptor agonist liraglutide on blood pressure profile, hydration, natriuresis in diabetic patients with severely impaired kidney function. Sci Rep. 2024;14:5002. 10.1038/s41598-024-55724-z. 38424466 PMC10904847

[51] Petersen KE, Rakipovski G, Raun K et al. Does glucagon-like peptide-1 ameliorate oxidative stress in diabetes? Evidence based on experimental and clinical studies. Curr Diabetes Rev. 2016;12:331–58. 10.2174/1573399812666150918150608. 26381142 PMC5101636

[52] Guarnotta V, Bianco MJ, Vigneri E et al. Effects of GLP-1 receptor agonists on myokine levels and pro-inflammatory cytokines in patients with type 2 diabetes mellitus. Nutr Metab Cardiovasc Dis. 2021;31:3193–201. 10.1016/j.numecd.2021.07.015. 34518091

[53] Ravic M, Srejovic I, Novakovic J et al. Effect of GLP-1 receptor agonist on ischemia reperfusion injury in rats with metabolic syndrome. Pharmaceuticals (Basel). 2024;17:525. 10.3390/ph17040525 38675485 PMC11053642

[54] J C, Me C, Mt C. Renoprotective mechanisms of glucagon-like peptide-1 receptor agonists. Diabetes Metab. 2025;51:101641. 40127835 10.1016/j.diabet.2025.101641

[55] Kanbay M, Copur S, Bakir CN et al. Glomerular hyperfiltration as a therapeutic target for CKD. Nephrol Dial Transplant. 2024;39:1228–38. 10.1093/ndt/gfae027. 38308513 PMC12086678

[56] Yao H, Zhang A, Li D et al. Comparative effectiveness of GLP-1 receptor agonists on glycaemic control, body weight, and lipid profile for type 2 diabetes: systematic review and network meta-analysis. BMJ. 2024;384:e076410. 10.1136/bmj-2023-076410. 38286487 PMC10823535

[57] Thomas MC, Coughlan MT, Cooper ME. The postprandial actions of GLP-1 receptor agonists: the missing link for cardiovascular and kidney protection in type 2 diabetes. Cell Metab. 2023;35:253–73. 10.1016/j.cmet.2023.01.004. 36754019

[58] Guo C, Huang T, Chen A et al. Glucagon-like peptide 1 improves insulin resistance in vitro through anti-inflammation of macrophages. Braz J Med Biol Res. 2016;49:e5826. 10.1590/1414-431x20165826. 27878229 PMC5188858

[59] Xu W, Sang YQ, Liu XK et al. Effect of glucagon-like peptide-1 receptor agonist on insulin secretion index and serum Wnt5a protein in patients with new-onset type 2 diabetes mellitus. J Diabetes Metab Disord. 2023;22:539–45. 10.1007/s40200-022-01175-0. 37255814 PMC10225441

[60] Lin YH, Lin CH, Huang YY et al. Regimen comprising GLP-1 receptor agonist and basal insulin can decrease the effect of food on glycemic variability compared to a pre-mixed insulin regimen. Eur J Med Res. 2022;27:273. 10.1186/s40001-022-00892-9. 36463197 PMC9719195

[61] Liu Y, Ruan B, Jiang H et al. The weight-loss effect of GLP-1RAs glucagon-like peptide-1 receptor agonists in non-diabetic individuals with overweight or obesity: a systematic review with meta-analysis and trial sequential analysis of randomized controlled trials. Am J Clin Nutr. 2023;118:614–26. 10.1016/j.ajcnut.2023.04.017. 37661106

[62] Dimitri P, Roth CL. Treatment of hypothalamic obesity with GLP-1 analogs. J Endocr Soc. 2024;9:bvae200. 10.1210/jendso/bvae200 39703362 PMC11655849

[63] Park JS, Kim KS, Choi HJ. Glucagon-like peptide-1 and hypothalamic regulation of satiation: cognitive and neural insights from human and animal studies. Diabetes Metab J. 2025;49:333–47. 10.4093/dmj.2025.0106. 40367985 PMC12086555

[64] Rivera FB, Lumbang GNO, Gaid DRM et al. Glucagon-like peptide-1 receptor agonists modestly reduced blood pressure among patients with and without diabetes mellitus: a meta-analysis and meta-regression. Diabetes Obes Metab. 2024;26:2209–28. 10.1111/dom.15529. 38505997

[65] Sun F, Wu S, Wang J et al. Effect of glucagon-like peptide-1 receptor agonists on lipid profiles among type 2 diabetes: a systematic review and network meta-analysis. Clin Ther. 2015;37:225–41.e8. 10.1016/j.clinthera.2014.11.008. 25554560

[66] Lee SA, Verhoeff R, Hullekes F et al. SGLT2 inhibitors and GLP-1 receptor agonists in kidney transplantation: a systematic review and meta-analysis. Transplantation. 2026;110:e217–e28. 10.1097/TP.0000000000005496. 40702593 PMC13725813

