# Supplementary material for: Clinical outcomes of glucagon-like peptide-1 receptor agonist therapy in kidney transplant recipients: a systematic review and meta-analysis
Source: Clin Kidney J. 2026 Mar 26;19(4):sfag105. doi: 10.1093/ckj/sfag105 (PMC13092978; doi:10.1093/ckj/sfag105)
Supplement: sfag105_Supplemental_File [file sfag105_Supplemental_File.docx]

**SUPPLEMENTAL FILE CONTENT**

- **Supplementary Table S1.** PRISMA 2020 checklist
- **Supplementary Table S2.** Database search strategy and keywords
- **Supplementary Table S3.** Risk of bias assessment using the Risk of Bias in Non-randomized Studies of Interventions (ROBINS-I) tool for included cohort studies
- **Supplementary Table S4.** Quality assessment of conference abstracts using the Newcastle–Ottawa Scale (NOS)
- **Supplementary Table S5.** Summary of findings (GRADE) for GLP-1RA therapy in kidney transplant recipients
- **Supplementary Table S6.** Summary of metabolic, renal, immunosuppressive, and safety outcomes of GLP-1 receptor agonists in kidney transplant recipients across included studies
- **Supplementary Table S7.** Gastrointestinal adverse events with GLP-1RA therapy in kidney transplant recipients
- **Supplementary Table S8.** Characteristics and key findings of abstracts and conference materials reporting GLP-1RA use in kidney transplant recipients **Supplementary Table S9.** Sensitivity of pooled estimates to meta-analytic model choice
- **Supplementary Figure S1.** Funnel plots to assess publication bias (meta-analyses with ≥10 studies). (A) HbA1c within-group analysis. (B) BMI within-group analysis

**Supplementary Table S1.** PRISMA 2020 checklist

| **Section and Topic** | **Item #** | **Checklist item** | **Location where item is reported** |
| --- | --- | --- | --- |
| **TITLE** | | |  |
| Title | 1 | Identify the report as a systematic review. | Page 1 |
| **ABSTRACT** | | |  |
| Abstract | 2 | See the PRISMA 2020 for Abstracts checklist. | Page 2 |
| **INTRODUCTION** | | |  |
| Rationale | 3 | Describe the rationale for the review in the context of existing knowledge. | Page 3-4 |
| Objectives | 4 | Provide an explicit statement of the objective(s) or question(s) the review addresses. | Page 4 |
| **METHODS** | | |  |
| Eligibility criteria | 5 | Specify the inclusion and exclusion criteria for the review and how studies were grouped for the syntheses. | Page 5-6 |
| Information sources | 6 | Specify all databases, registers, websites, organisations, reference lists and other sources searched or consulted to identify studies. Specify the date when each source was last searched or consulted. | Page 5 |
| Search strategy | 7 | Present the full search strategies for all databases, registers and websites, including any filters and limits used. | Page 5; Supplementary Table S2 |
| Selection process | 8 | Specify the methods used to decide whether a study met the inclusion criteria of the review, including how many reviewers screened each record and each report retrieved, whether they worked independently, and if applicable, details of automation tools used in the process. | Page 6 |
| Data collection process | 9 | Specify the methods used to collect data from reports, including how many reviewers collected data from each report, whether they worked independently, any processes for obtaining or confirming data from study investigators, and if applicable, details of automation tools used in the process. | Page 6 |
| Data items | 10a | List and define all outcomes for which data were sought. Specify whether all results that were compatible with each outcome domain in each study were sought (e.g. for all measures, time points, analyses), and if not, the methods used to decide which results to collect. | Page 6 |
|  | 10b | List and define all other variables for which data were sought (e.g. participant and intervention characteristics, funding sources). Describe any assumptions made about any missing or unclear information. | Page 6 |
| Study risk of bias assessment | 11 | Specify the methods used to assess risk of bias in the included studies, including details of the tool(s) used, how many reviewers assessed each study and whether they worked independently, and if applicable, details of automation tools used in the process. | Page 7 |
| Effect measures | 12 | Specify for each outcome the effect measure(s) (e.g. risk ratio, mean difference) used in the synthesis or presentation of results. | Page 6 |
| Synthesis methods | 13a | Describe the processes used to decide which studies were eligible for each synthesis (e.g. tabulating the study intervention characteristics and comparing against the planned groups for each synthesis (item #5)). | Page 6 |
|  | 13b | Describe any methods required to prepare the data for presentation or synthesis, such as handling of missing summary statistics, or data conversions. | Page 6 |
|  | 13c | Describe any methods used to tabulate or visually display results of individual studies and syntheses. | Page 6; Supplementary Tables S6–S10 |
|  | 13d | Describe any methods used to synthesize results and provide a rationale for the choice(s). If meta-analysis was performed, describe the model(s), method(s) to identify the presence and extent of statistical heterogeneity, and software package(s) used. | Page 6-7 |
|  | 13e | Describe any methods used to explore possible causes of heterogeneity among study results (e.g. subgroup analysis, meta-regression). | Page 6-7 |
|  | 13f | Describe any sensitivity analyses conducted to assess robustness of the synthesized results. | Page 6 |
| Reporting bias assessment | 14 | Describe any methods used to assess risk of bias due to missing results in a synthesis (arising from reporting biases). | Page 7 |
| Certainty assessment | 15 | Describe any methods used to assess certainty (or confidence) in the body of evidence for an outcome. | Page 7 |
| **RESULTS** | | |  |
| Study selection | 16a | Describe the results of the search and selection process, from the number of records identified in the search to the number of studies included in the review, ideally using a flow diagram. | Page 7 |
|  | 16b | Cite studies that might appear to meet the inclusion criteria, but which were excluded, and explain why they were excluded. | Page 7 |
| Study characteristics | 17 | Cite each included study and present its characteristics. | Table 1; Pages 7-8 |
| Risk of bias in studies | 18 | Present assessments of risk of bias for each included study. | Supplementary Tables S3 and S4 |
| Results of individual studies | 19 | For all outcomes, present, for each study: (a) summary statistics for each group (where appropriate) and (b) an effect estimate and its precision (e.g. confidence/credible interval), ideally using structured tables or plots. | Tables 1-3; Figures 2-10 |
| Results of syntheses | 20a | For each synthesis, briefly summarise the characteristics and risk of bias among contributing studies. | Page 7; Supplementary Table S3 |
|  | 20b | Present results of all statistical syntheses conducted. If meta-analysis was done, present for each the summary estimate and its precision (e.g. confidence/credible interval) and measures of statistical heterogeneity. If comparing groups, describe the direction of the effect. | Pages 7-15; Figures 2-10 |
|  | 20c | Present results of all investigations of possible causes of heterogeneity among study results. | Pages 7-15 |
|  | 20d | Present results of all sensitivity analyses conducted to assess the robustness of the synthesized results. | Pages 7-15 |
| Reporting biases | 21 | Present assessments of risk of bias due to missing results (arising from reporting biases) for each synthesis assessed. | Page 16; Supplementary Figure S1 |
| Certainty of evidence | 22 | Present assessments of certainty (or confidence) in the body of evidence for each outcome assessed. | Page 16; Supplementary Table S5 |
| **DISCUSSION** | | |  |
| Discussion | 23a | Provide a general interpretation of the results in the context of other evidence. | Pages 16-19 |
|  | 23b | Discuss any limitations of the evidence included in the review. | Page 19 |
|  | 23c | Discuss any limitations of the review processes used. | Page 19 |
|  | 23d | Discuss implications of the results for practice, policy, and future research. | Page 19 |
| **OTHER INFORMATION** | | |  |
| Registration and protocol | 24a | Provide registration information for the review, including register name and registration number, or state that the review was not registered. | Page 4 |
|  | 24b | Indicate where the review protocol can be accessed, or state that a protocol was not prepared. | Page 4 |
|  | 24c | Describe and explain any amendments to information provided at registration or in the protocol. | Page 4 |
| Support | 25 | Describe sources of financial or non-financial support for the review, and the role of the funders or sponsors in the review. | Page 20 |
| Competing interests | 26 | Declare any competing interests of review authors. | Page 20 |
| Availability of data, code and other materials | 27 | Report which of the following are publicly available and where they can be found: template data collection forms; data extracted from included studies; data used for all analyses; analytic code; any other materials used in the review. | Page 20 |

**Supplementary Table S2.** Database search strategy and keywords

| **PubMed (n=428)** |
| --- |
| 3 ("Kidney Transplantation"[Mesh] OR "Tissue and Organ Procurement"[Mesh] OR "kidney transplant*" OR "renal transplant*" OR "kidney graft" OR "renal graft" OR "renal allograft" OR "kidney allograft" OR "post-transplant kidney" OR "renal transplantation" OR "kidney transplantation") AND ("Glucagon-Like Peptide 1 Receptor Agonists"[Mesh] OR "Incretins"[Mesh] OR "Hypoglycemic Agents"[Mesh] OR "Glucagon-Like Peptide 1"[Mesh] OR "GLP-1 receptor agonist*" OR "GLP-1 RA" OR "GLP1RA" OR semaglutide OR liraglutide OR dulaglutide OR exenatide OR albiglutide OR lixisenatide OR efpeglenatide OR Ozempic OR Victoza OR Trulicity OR Byetta OR Lyxumia OR "dual incretin agonist*" OR "GLP-1/GIP agonist*" OR "GLP-1 and GIP receptor agonist*" OR tirzepatide OR LY3298176 OR Mounjaro OR "triple agonist*" OR "GLP-1/GIP/glucagon agonist*" OR SAR441255 OR LY3437943 OR retatrutide OR "GIP/GLP-1 receptor agonist*" OR "GLP-1/glucagon receptor agonist*" OR "glucagon/GLP-1 dual agonist*" OR "GIP/GLP-1 agonist*" OR "dual incretin receptor agonist*" OR "triagonist*" OR "dual incretin agonist*" OR "GLP-1 analog*" OR "GLP1 receptor agonist*" OR "GLP-1RA" OR "incretin mimetic*" OR Cotadutide OR "antidiabetic agent*" OR "incretin-based therap*") Publication Date ("Kidney Transplantation"[MeSH Terms] OR "Tissue and Organ Procurement"[MeSH Terms] OR "kidney transplant*"[All Fields] OR "renal transplant*"[All Fields] OR "kidney graft"[All Fields] OR "renal graft"[All Fields] OR "renal allograft"[All Fields] OR "kidney allograft"[All Fields] OR "post-transplant kidney"[All Fields] OR "renal transplantation"[All Fields] OR "Kidney Transplantation"[All Fields]) AND ("Glucagon-Like Peptide 1 Receptor Agonists"[MeSH Terms] OR "Incretins"[MeSH Terms] OR "Hypoglycemic Agents"[MeSH Terms] OR "Glucagon-Like Peptide 1"[MeSH Terms] OR "glp 1 receptor agonist*"[All Fields] OR "GLP-1 RA"[All Fields] OR "GLP1RA"[All Fields] OR ("semaglutide"[Supplementary Concept] OR "semaglutide"[All Fields]) OR ("liraglutid"[All Fields] OR "liraglutide"[Supplementary Concept] OR "liraglutide"[All Fields] OR "liraglutide"[MeSH Terms] OR "liraglutide s"[All Fields]) OR ("dulaglutide"[Supplementary Concept] OR "dulaglutide"[All Fields]) OR ("exenatide"[Supplementary Concept] OR "exenatide"[All Fields] OR "exenatide"[MeSH Terms] OR "exenatide s"[All Fields]) OR ("rglp 1 protein"[Supplementary Concept] OR "rglp 1 protein"[All Fields] OR "albiglutide"[All Fields]) OR ("lixisenatide"[Supplementary Concept] OR "lixisenatide"[All Fields]) OR ("efpeglenatide"[Supplementary Concept] OR "efpeglenatide"[All Fields]) OR ("semaglutide"[Supplementary Concept] OR "semaglutide"[All Fields] OR "ozempic"[All Fields]) OR ("liraglutid"[All Fields] OR "liraglutide"[Supplementary Concept] OR "liraglutide"[All Fields] OR "liraglutide"[MeSH Terms] OR "victoza"[All Fields] OR "liraglutide s"[All Fields]) OR ("dulaglutide"[Supplementary Concept] OR "dulaglutide"[All Fields] OR "trulicity"[All Fields]) OR ("exenatide"[Supplementary Concept] OR "exenatide"[All Fields] OR "byetta"[All Fields] OR "exenatide"[MeSH Terms] OR "exenatide s"[All Fields]) OR ("lixisenatide"[Supplementary Concept] OR "lixisenatide"[All Fields] OR "lyxumia"[All Fields]) OR "dual incretin agonist*"[All Fields] OR "glp 1 gip agonist*"[All Fields] OR "glp 1 and gip receptor agonist*"[All Fields] OR ("tirzepatide"[Supplementary Concept] OR "tirzepatide"[All Fields] OR "tirzepatide"[MeSH Terms]) OR ("tirzepatide"[Supplementary Concept] OR "tirzepatide"[All Fields] OR "ly3298176"[All Fields] OR "tirzepatide"[MeSH Terms]) OR ("tirzepatide"[Supplementary Concept] OR "tirzepatide"[All Fields] OR "mounjaro"[All Fields] OR "tirzepatide"[MeSH Terms]) OR "triple agonist*"[All Fields] OR "glp 1 gip glucagon agonist*"[All Fields] OR "SAR441255"[All Fields] OR ("retatrutide"[Supplementary Concept] OR "retatrutide"[All Fields] OR "ly3437943"[All Fields]) OR ("retatrutide"[Supplementary Concept] OR "retatrutide"[All Fields]) OR "gip glp 1 receptor agonist*"[All Fields] OR "glp 1 glucagon receptor agonist*"[All Fields] OR "glucagon glp 1 dual agonist*"[All Fields] OR "gip glp 1 agonist*"[All Fields] OR "dual incretin receptor agonist*"[All Fields] OR "triagonist*"[All Fields] OR "dual incretin agonist*"[All Fields] OR "glp 1 analog*"[All Fields] OR "glp1 receptor agonist*"[All Fields] OR "GLP-1RA"[All Fields] OR "incretin mimetic*"[All Fields] OR ("cotadutide"[Supplementary Concept] OR "cotadutide"[All Fields]) OR "antidiabetic agent*"[All Fields] OR "incretin based therap*"[All Fields]) 428  2 "Glucagon-Like Peptide 1 Receptor Agonists"[Mesh] OR "Incretins"[Mesh] OR "Hypoglycemic Agents"[Mesh] OR "Glucagon-Like Peptide 1"[Mesh] OR "GLP-1 receptor agonist*" OR "GLP-1 RA" OR "GLP1RA" OR semaglutide OR liraglutide OR dulaglutide OR exenatide OR albiglutide OR lixisenatide OR efpeglenatide OR Ozempic OR Victoza OR Trulicity OR Byetta OR Lyxumia OR "dual incretin agonist*" OR "GLP-1/GIP agonist*" OR "GLP-1 and GIP receptor agonist*" OR tirzepatide OR LY3298176 OR Mounjaro OR "triple agonist*" OR "GLP-1/GIP/glucagon agonist*" OR SAR441255 OR LY3437943 OR retatrutide OR "GIP/GLP-1 receptor agonist*" OR "GLP-1/glucagon receptor agonist*" OR "glucagon/GLP-1 dual agonist*" OR "GIP/GLP-1 agonist*" OR "dual incretin receptor agonist*" OR "triagonist*" OR "dual incretin agonist*" OR "GLP-1 analog*" OR "GLP1 receptor agonist*" OR "GLP-1RA" OR "incretin mimetic*" OR Cotadutide OR "antidiabetic agent*" OR "incretin-based therap*" Publication Date "Glucagon-Like Peptide 1 Receptor Agonists"[MeSH Terms] OR "Incretins"[MeSH Terms] OR "Hypoglycemic Agents"[MeSH Terms] OR "Glucagon-Like Peptide 1"[MeSH Terms] OR "glp 1 receptor agonist*"[All Fields] OR "GLP-1 RA"[All Fields] OR "GLP1RA"[All Fields] OR "semaglutide"[Supplementary Concept] OR "semaglutide"[All Fields] OR "liraglutid"[All Fields] OR "liraglutide"[Supplementary Concept] OR "liraglutide"[All Fields] OR "liraglutide"[MeSH Terms] OR "liraglutide s"[All Fields] OR "dulaglutide"[Supplementary Concept] OR "dulaglutide"[All Fields] OR "exenatide"[Supplementary Concept] OR "exenatide"[All Fields] OR "exenatide"[MeSH Terms] OR "exenatide s"[All Fields] OR "rglp 1 protein"[Supplementary Concept] OR "rglp 1 protein"[All Fields] OR "albiglutide"[All Fields] OR "lixisenatide"[Supplementary Concept] OR "lixisenatide"[All Fields] OR "efpeglenatide"[Supplementary Concept] OR "efpeglenatide"[All Fields] OR "semaglutide"[Supplementary Concept] OR "semaglutide"[All Fields] OR "ozempic"[All Fields] OR "liraglutid"[All Fields] OR "liraglutide"[Supplementary Concept] OR "liraglutide"[All Fields] OR "liraglutide"[MeSH Terms] OR "victoza"[All Fields] OR "liraglutide s"[All Fields] OR "dulaglutide"[Supplementary Concept] OR "dulaglutide"[All Fields] OR "trulicity"[All Fields] OR "exenatide"[Supplementary Concept] OR "exenatide"[All Fields] OR "byetta"[All Fields] OR "exenatide"[MeSH Terms] OR "exenatide s"[All Fields] OR "lixisenatide"[Supplementary Concept] OR "lixisenatide"[All Fields] OR "lyxumia"[All Fields] OR "dual incretin agonist*"[All Fields] OR "glp 1 gip agonist*"[All Fields] OR "glp 1 and gip receptor agonist*"[All Fields] OR "tirzepatide"[Supplementary Concept] OR "tirzepatide"[All Fields] OR "tirzepatide"[MeSH Terms] OR "tirzepatide"[Supplementary Concept] OR "tirzepatide"[All Fields] OR "ly3298176"[All Fields] OR "tirzepatide"[MeSH Terms] OR "tirzepatide"[Supplementary Concept] OR "tirzepatide"[All Fields] OR "mounjaro"[All Fields] OR "tirzepatide"[MeSH Terms] OR "triple agonist*"[All Fields] OR "glp 1 gip glucagon agonist*"[All Fields] OR "SAR441255"[All Fields] OR "retatrutide"[Supplementary Concept] OR "retatrutide"[All Fields] OR "ly3437943"[All Fields] OR "retatrutide"[Supplementary Concept] OR "retatrutide"[All Fields] OR "gip glp 1 receptor agonist*"[All Fields] OR "glp 1 glucagon receptor agonist*"[All Fields] OR "glucagon glp 1 dual agonist*"[All Fields] OR "gip glp 1 agonist*"[All Fields] OR "dual incretin receptor agonist*"[All Fields] OR "triagonist*"[All Fields] OR "dual incretin agonist*"[All Fields] OR "glp 1 analog*"[All Fields] OR "glp1 receptor agonist*"[All Fields] OR "GLP-1RA"[All Fields] OR "incretin mimetic*"[All Fields] OR "cotadutide"[Supplementary Concept] OR "cotadutide"[All Fields] OR "antidiabetic agent*"[All Fields] OR "incretin based therap*"[All Fields] 115,032  1 "Kidney Transplantation"[Mesh] OR "Tissue and Organ Procurement"[Mesh] OR "kidney transplant*" OR "renal transplant*" OR "kidney graft" OR "renal graft" OR "renal allograft" OR "kidney allograft" OR "post-transplant kidney" OR "renal transplantation" OR "kidney transplantation" Publication Date "Kidney Transplantation"[MeSH Terms] OR "Tissue and Organ Procurement"[MeSH Terms] OR "kidney transplant*"[All Fields] OR "renal transplant*"[All Fields] OR "kidney graft"[All Fields] OR "renal graft"[All Fields] OR "renal allograft"[All Fields] OR "kidney allograft"[All Fields] OR "post-transplant kidney"[All Fields] OR "renal transplantation"[All Fields] OR "Kidney Transplantation"[All Fields] 166,306 |
| **Scopus (n=978)** |
| TITLE-ABS-KEY ( "kidney transplant*" OR "renal transplant*" OR "kidney graft" OR "renal graft" OR "renal allograft" OR "kidney allograft" OR "post-transplant kidney" OR "renal transplantation" OR "kidney transplantation" ) AND TITLE-ABS-KEY ( "GLP-1 receptor agonist*" OR "GLP-1 RA" OR "GLP1RA" OR semaglutide OR liraglutide OR dulaglutide OR exenatide OR albiglutide OR lixisenatide OR efpeglenatide OR Ozempic OR Victoza OR Trulicity OR Byetta OR Lyxumia OR "dual incretin agonist*" OR "GLP-1/GIP agonist*" OR "GLP-1 and GIP receptor agonist*" OR tirzepatide OR LY3298176 OR Mounjaro OR "triple agonist*" OR "GLP-1/GIP/glucagon agonist*" OR SAR441255 OR LY3437943 OR retatrutide OR "GIP/GLP-1 receptor agonist*" OR "GLP-1/glucagon receptor agonist*" OR "glucagon/GLP-1 dual agonist*" OR "GIP/GLP-1 agonist*" OR "dual incretin receptor agonist*" OR "triagonist*" OR "dual incretin agonist*" OR "GLP-1 analog*" OR "GLP1 receptor agonist*" OR "GLP-1RA" OR "incretin mimetic*" OR Cotadutide OR "antidiabetic agent*" OR "incretin-based therap*" )  978 |
| **Web of Science (n=125)** |
| 1: TS=("kidney transplant*" OR "renal transplant*" OR "kidney graft" OR "renal graft" OR "renal allograft" OR "kidney allograft" OR "post-transplant kidney" OR "renal transplantation" OR "kidney transplantation" ) Date Run: Mon Sep 29 2025 12:37:33 GMT+0300 (GMT+03:00) Results: 154023  2: TS=("GLP-1 receptor agonist*" OR "GLP-1 RA" OR "GLP1RA" OR semaglutide OR liraglutide OR dulaglutide OR exenatide OR albiglutide OR lixisenatide OR efpeglenatide OR Ozempic OR Victoza OR Trulicity OR Byetta OR Lyxumia OR "dual incretin agonist*" OR "GLP-1/GIP agonist*" OR "GLP-1 and GIP receptor agonist*" OR tirzepatide OR LY3298176 OR Mounjaro OR "triple agonist*" OR "GLP-1/GIP/glucagon agonist*" OR SAR441255 OR LY3437943 OR retatrutide OR "GIP/GLP-1 receptor agonist*" OR "GLP-1/glucagon receptor agonist*" OR "glucagon/GLP-1 dual agonist*" OR "GIP/GLP-1 agonist*" OR "dual incretin receptor agonist*" OR "triagonist*" OR "dual incretin agonist*" OR "GLP-1 analog*" OR "GLP1 receptor agonist*" OR "GLP-1RA" OR "incretin mimetic*" OR Cotadutide OR "antidiabetic agent*" OR "incretin-based therap*") Date Run: Mon Sep 29 2025 12:37:45 GMT+0300 (GMT+03:00) Results: 29357  3: #2 AND #1 Date Run: Mon Sep 29 2025 12:37:50 GMT+0300 (GMT+03:00) Results: 125 |
| **Cochrane Library (n=71)** |
| kidney transplant* OR renal transplant* OR "kidney graft" OR "renal graft" OR "renal allograft" OR "kidney allograft" OR "post-transplant kidney" OR "renal transplantation" OR "kidney transplantation" in Title Abstract Keyword AND GLP-1 receptor agonist* OR "GLP-1 RA" OR "GLP1RA" OR semaglutide OR liraglutide OR dulaglutide OR exenatide OR albiglutide OR lixisenatide OR efpeglenatide OR Ozempic OR Victoza OR Trulicity OR Byetta OR Lyxumia OR dual incretin agonist* OR GLP-1 GIP agonist* OR GLP-1 and GIP receptor agonist* OR tirzepatide OR LY3298176 OR Mounjaro OR triple agonist* OR GLP-1 GIP glucagon agonist* OR SAR441255 OR LY3437943 OR retatrutide OR GIP GLP-1 receptor agonist* OR GLP-1 glucagon receptor agonist* OR glucagon GLP-1 dual agonist* OR GIP GLP-1 agonist* OR dual incretin receptor agonist* OR triagonist* OR dual incretin agonist* OR GLP-1 analog* OR GLP1 receptor agonist* OR "GLP-1RA" OR incretin mimetic* OR Cotadutide OR antidiabetic agent* OR incretin-based therap* in Title Abstract Keyword - (Word variations have been searched)  71 |
| **Ovid MEDLINE (n=103)** |
| Ovid MEDLINE(R) and Epub Ahead of Print, In-Process, In-Data-Review & Other Non-Indexed Citations, Daily and Versions <1946 to September 26, 2025>  1 (("kidney transplant*" or "renal transplant*" or "kidney graft" or "renal graft" or "renal allograft" or "kidney allograft" or "post-transplant kidney" or "renal transplantation" or "kidney transplantation") and ("GLP-1 receptor agonist*" or "GLP-1 RA" or "GLP1RA" or semaglutide or liraglutide or dulaglutide or exenatide or albiglutide or lixisenatide or efpeglenatide or Ozempic or Victoza or Trulicity or Byetta or Lyxumia or "dual incretin agonist*" or "GLP-1 GIP agonist*" or "GLP-1 and GIP receptor agonist*" or tirzepatide or LY3298176 or Mounjaro or "triple agonist*" or "GLP-1 GIP glucagon agonist*" or SAR441255 or LY3437943 or retatrutide or "GIP GLP-1 receptor agonist*" or "GLP-1 glucagon receptor agonist*" or "glucagon GLP-1 dual agonist*" or "GIP GLP-1 agonist*" or "dual incretin receptor agonist*" or "triagonist*" or "dual incretin agonist*" or "GLP-1 analog*" or "GLP1 receptor agonist*" or "GLP-1RA" or "incretin mimetic*" or Cotadutide or "antidiabetic agent*" or "incretin-based therap*")).af. 103 |
| **Total Records: 1705** |

**Supplementary Table S3.** Risk of bias assessment using the Risk of Bias in Non-randomized Studies of Interventions (ROBINS-I) tool for included cohort studies

| ***First author, year*** | ***Bias in confounding*** | ***Bias in selection*** | ***Bias in classification of interventions*** | ***Bias due to deviations from intended interventions*** | ***Bias due to missing data*** | ***Bias in measurement outcome*** | ***Bias in selection of reported Result*** | ***Overall risk of bias judgement*** |  |  |
| --- | --- | --- | --- | --- | --- | --- | --- | --- | --- | --- |
| *Cohen et al., 2025* | Low | Moderate | Low | Low | Moderate | Low | Low | Moderate |  |  |
| *Freitas et al., 2024* | Moderate | Moderate | Low | Moderate | Low | Low | Low | Moderate |  |  |
| González et al., 2021 | Low | Low | Low | Low | Low | Moderate | Low | Moderate |  |  |
| *Kahwaji et al., 2024* | Moderate | Moderate | Low | Moderate | Moderate | Low | Low | Moderate |  |  |
| *Kim et al., 2021* | Low | Low | Low | Low | Low | Moderate | Low | Moderate |  |  |
| *Kukla et al., 2020* | Low | Low | Low | Low | Low | Moderate | Low | Moderate |  |  |
| *Lin et al., 2025* | Low | Low | Low | Moderate | Low | Low | Low | Moderate |  |  |
| *Liou et al., 2018* | Moderate | Moderate | Low | Moderate | Low | Moderate | Low | Moderate |  |  |
| *Mahmoud et al., 2023* | Low | Moderate | Low | Moderate | Low | Low | Low | Moderate |  |  |
| *Mahzari et al., 2024* | Moderate | Low | Low | Low | Moderate | Low | Low | Moderate |  |  |
| *Mallik et al., 2023* | Moderate | Low | Low | Low | Moderate | Moderate | Low | Moderate |  |  |
| *Orandi et al., 2025* | Low | Low | Low | Low | Low | Low | Low | Low |  |  |
| *Sahi et al., 2025* | Low | Moderate | Low | Moderate | Moderate | Low | Low | Moderate |  |  |
| *Sato et al., 2023* | Low | Low | Low | Low | Moderate | Moderate | Low | Moderate |  |  |
| *Vigara et al., 2022* | Moderate | Moderate | Low | Moderate | Moderate | Low | Low | Moderate |  |  |
| *Vigara et al., 2024* | Moderate | Moderate | Low | Moderate | Moderate | Low | Low | Moderate |  |  |
| *Zelada et al., 2025* | Low | Moderate | Low | Low | Low | Low | Low | Moderate |  |  |
|  |  |  |  |  |  |  |  |  |  |  |

**Supplementary Table S4.** Quality assessment of conference abstracts using the Newcastle–Ottawa Scale (NOS)

|  | **Selection** | | | | **Comparability** | **Outcome** | | |  |  |
| --- | --- | --- | --- | --- | --- | --- | --- | --- | --- | --- |
| Author name, year | Representativeness of exposed cohorts | Selection of non-exposed cohort | Ascertainment of exposure | Demonstration that outcome was not present at start | Study controlled for key confounders | Assessment of outcome | Sufficient follow up duration | Adequacy of follow up | Total stars | Overall bias |
| *Acosta et al. (2025)* | * | * | * | * | * | * | * | * | 8 | Low |
| *Attallah et al. (2022)* | * |  | * | * |  | * | * | * | 6 | Moderate |
| *Lim et al. (2025)* | * |  | * | * |  | * | * | * | 6 | Moderate |
| *Martin et al. (2025)* | * |  | * | * |  | * | * | * | 6 | Moderate |
| *Miura et al. (2025)* | * | * | * | * | * | * | * | * | 8 | Low |
| *Pham et al. (2025)* | * |  | * | * |  | * | * | * | 6 | Moderate |
| *Sridhar et al. (2023)* | * | * | * | * | * | * | * | * | 8 | Low |

**Supplementary Table S5.** Summary of findings table: GRADE evaluation of GLP-1RA in kidney transplant recipients

| ***Outcome*** | ***No. of Studies (Patients)*** | ***Study Design*** | ***Risk of Bias*** | ***Inconsistency*** | ***Indirectness*** | ***Imprecision*** | ***Publication Bias*** | ***Overall Certainty*** | ***Rationale / Comments*** |  |  |
| --- | --- | --- | --- | --- | --- | --- | --- | --- | --- | --- | --- |
| ***All-cause mortality*** | | 4 (>21,000) | Observational cohorts | Moderate | Moderate (I² = 76%) | Low | Low (CI narrow) | Low | **Moderate** | Large consistent effect across cohorts; adjusted HR ≈ 0.5. |  |
| ***Major adverse cardiovascular events (MACE)*** | | | 3 (≈ 20,000) | Observational cohorts | Moderate | Low (I² = 0%) | Low | Low | Low | **Moderate** | Consistent cardioprotection in large studies. |
| ***eGFR change (3–24 mo)*** | | | 16 (≈ 3,000) | Observational cohorts | Moderate | Moderate (0–55%) | Low | Moderate | Low | **Low** | Stable or improved renal function; heterogeneity from differing follow-up and baseline CKD stage. |
| ***Graft loss / MAKE / rejection*** | | | 6 (>20,000) | Observational cohorts | Moderate | Low | Low | Moderate | Low | **Low** | Reduced composite renal events; consistency but limited RCTs. |
| ***HbA1c reduction*** | | | 13 (≈ 2,000) | Observational cohorts | Moderate | High (I² = 94%) | Low | Moderate | Possible (small-study effect p = 0.005) | **Low** | Consistent direction of effect but wide heterogeneity. |
| ***BMI reduction*** | | | 12 (≈ 1,800) | Observational cohorts | Moderate | High (I² = 86%) | Low | Moderate | Possible (p = 0.0004) | **Low** | Significant weight loss with substantial heterogeneity. |
| ***Insulin requirement reduction*** | | | 4 (≈ 200) | Observational cohorts | Moderate | High (I² = 77%) | Low | High | Low | **Low** | Few small studies; wide CIs. |
| ***Tacrolimus levels (6–12 mo)*** | | | 6 (≈ 500) | Observational cohorts | Low | Low (I² ≤ 45%) | Low | Low | Low | **Moderate** | Consistent neutrality; narrow CIs. |
| ***uACR / proteinuria change*** | | | 3 (≈ 200) | Observational cohorts | Moderate | Low (I² = 0%) | Low | Moderate | Low | **Low** | Small sample size but consistent improvement. |
| ***GI adverse events*** | | | 9 (≈ 800) | Observational cohorts | Moderate | Low | Low | Low | Low | **Moderate** | Mild, self-limited nausea common; few discontinuations. |
| ***Pancreatitis / biliary events*** | | | 6 (>18,000) | Observational cohorts | Moderate | Low | Low | Moderate | Low | **Moderate** | Consistent neutrality after insulin adjustment. |
| ***Urogenital and opportunistic infections*** | | | 6 (≈ 600) | Observational cohorts | Moderate | Low | Low | Moderate | Low | **Moderate** | No signal of increased infection risk. |
| ***Discontinuation rate*** | | | 11 (≈ 1,200) | Observational cohorts | Moderate | Moderate | Low | Moderate | Low | **Low** | Varied by tolerance and access; mostly GI-related. |

**Supplementary Table S6.** Summary of metabolic, renal, immunosuppressive, and safety outcomes of GLP-1 receptor agonists in kidney transplant recipients across included studies

| **First author, year** | **Sample size** | **Study design** | **Metabolic outcomes** | **Renal outcomes** | **Immunosuppressive outcomes** | **Safety outcomes** | **Key conclusions** |
| --- | --- | --- | --- | --- | --- | --- | --- |
| Cohen et al., 2025 | 272 (136 GLP-1RA, 136 control) | RC | HbA1c decreased from 7.8 ± 1.4% to 7.3 ± 1.0% (p=0.01); BMI decreased −0.9 kg/m² (p=0.01) | eGFR change +0.6 vs −1.4 mL/min/1.73 m² (p=0.20); composite renal outcome HR 0.49 | Tacrolimus levels unchanged | Lower biliopancreatic adverse events vs control | GLP-1RA improved glycemic control and reduced composite renal outcome risk |
| Freitas et al., 2024 | 64 | RC | Mean HbA1c decreased −0.45% (p=0.002); BMI decreased −0.5 kg/m² (p=0.03) | Serum creatinine 1.53→1.41 mg/dL (p=0.30); proteinuria unchanged | Not reported | Minimal hypoglycemia; no pancreatitis reported | GLP-1RA improved metabolic control without adverse renal effects |
| González et al., 2021 | 15 | RC | Significant weight reduction; HbA1c decreased but not statistically significant | Serum creatinine 1.30→1.30 mg/dL (p=0.43); proteinuria unchanged | Tacrolimus levels stable throughout follow-up | One discontinuation due to weakness; no rejection episodes | GLP-1RA safe and effective for metabolic control |
| Kahwaji et al., 2025 | 185 | RC | HbA1c significantly decreased; BMI significantly reduced | eGFR 57→59.6 mL/min/1.73 m² (p=0.40); no graft failure | Tacrolimus levels stable | No graft failure events attributable to GLP-1RA | GLP-1RA improved metabolic profile with preserved graft function |
| Kim et al., 2021 | 37 | RC | Weight decreased −4.9 kg (p<0.001); HbA1c comparable to prandial insulin | Renal function remained stable during follow-up | Tacrolimus levels unchanged | Two patients discontinued therapy due to gastrointestinal symptoms | Dulaglutide effective alternative to insulin with acceptable safety |
| Kukla et al., 2020 | 17 | RC | Reduced insulin dose requirements; modest weight reduction observed | eGFR remained stable over study period | Tacrolimus dose and levels stable | Discontinuation rate 29%; gastrointestinal intolerance most common | GLP-1RA safe and effective adjunct therapy |
| Lin et al., 2025 | 6,594 matched pairs | RC | Weight and glycemic improvements observed | Mortality HR 0.39 (95% CI 0.31–0.50); MAKE HR 0.66 (95% CI 0.58–0.75) | Not reported | Higher gastrointestinal adverse event rates; no increase in pancreatitis | GLP-1RA associated with improved survival and renal outcomes |
| Liou et al., 2018 | 7 | RC | HbA1c significantly decreased (p<0.05); weight decreased | eGFR significantly improved (p=0.024); proteinuria decreased (not statistically significant) | Tacrolimus levels unchanged | Two discontinuations due to gastrointestinal intolerance | GLP-1RA improved glycemic and renal parameters safely |
| Mahmoud et al., 2023 | 111 | RC | HbA1c and BMI significantly decreased | eGFR stable; UACR significantly reduced | Tacrolimus levels stable | Urinary tract infection most frequent adverse event; no severe safety signals | GLP-1RA improved metabolic and albuminuria outcomes |
| Mahzari et al., 2024 | 39 | RC | HbA1c decreased by approximately 1%; weight significantly reduced | eGFR remained stable | Tacrolimus levels unchanged in 95% of patients | Therapy well tolerated with no major adverse events | Semaglutide effective and safe in kidney transplant recipients |
| Mallik et al., 2023 | 23 | RC | HbA1c significantly decreased; weight reduction observed | eGFR stable; no significant deterioration | Tacrolimus levels modestly decreased without clinical consequence | Three patients discontinued therapy due to gastrointestinal intolerance | GLP-1RA safe and effective metabolic therapy |
| Orandi et al., 2025 | 18,016 | RC | Metabolic outcomes not reported | Death-censored graft loss aSHR 0.51 (95% CI 0.36–0.71); mortality HR 0.69 (95% CI 0.55–0.86) | Not reported | Increased diabetic retinopathy risk; pancreatitis risk not increased | GLP-1RA associated with improved graft and patient survival |
| Sahi et al., 2025 | 2,171 | RC | HbA1c and BMI significantly reduced | Slower eGFR decline; reduced albuminuria | Not reported | GI discontinuation 5.4%; pancreatitis rare | GLP-1RA associated with improved renal trajectory |
| Sato et al., 2023 | 146 | RC | HbA1c remained stable | Reduced risk of sustained eGFR decline OR 0.105 | Not reported | No significant safety concerns reported | GLP-1RA associated with renal protective effects |
| Vigara et al., 2022 | 50 | RC | HbA1c significantly decreased; weight significantly decreased | eGFR improved; proteinuria significantly reduced | Tacrolimus levels unchanged | No acute rejection episodes; no dnDSA development | GLP-1RA safe with improved renal and metabolic outcomes |
| Vigara et al., 2024 | 96 | RC | HbA1c significantly reduced; weight and lipid profile improved | eGFR stable; proteinuria decreased | Tacrolimus levels unchanged | No major adverse events observed | GLP-1RA improved metabolic and renal parameters safely |
| Zelada et al., 2025 | 50 | RC | Weight significantly reduced; insulin requirements decreased | eGFR improved; proteinuria reduced | Tacrolimus levels stable | No therapy discontinuations due to adverse effects | GLP-1RA safe and effective in kidney transplant recipients |
|  |  |  |  |  |  |  |  |

**Abbreviations:** aHR, adjusted hazard ratio; aSHR, adjusted subdistribution hazard ratio; BMI, body mass index; CI, confidence interval; dnDSA, de novo donor-specific antibodies; eGFR, estimated glomerular filtration rate; GI, gastrointestinal; GLP-1RA, glucagon-like peptide-1 receptor agonist; HbA1c, glycated hemoglobin; HR, hazard ratio; MAKE, major adverse kidney events; NS, not statistically significant; OR, odds ratio; RC, retrospective cohort; SD, standard deviation; UACR, urine albumin-to-creatinine ratio.

**Supplementary Table S7.** Gastrointestinal adverse events with GLP-1RA therapy in kidney transplant recipients

| **Study** | **Year** | **n** | **GI adverse events reported** | **GI-related discontinuation** | **Notes** |
| --- | --- | --- | --- | --- | --- |
| *Cohen et al.* | 2025 | 136 | NA | NA | Not specified |
| *Freitas et al.* | 2024 | 64 | NA | NA | Not specified |
| *González et al.* | 2021 | 13 | NA | NA | Not specified |
| *Kahwaji et al.* | 2025 | 185 | Mild nausea, early satiety | None | No severe GI AEs |
| *Kim et al.* | 2021 | 37 | Mild nausea/decreased appetite in few | None | No hospitalizations |
| *Kukla et al.* | 2020 | 17 | 3 pts (14%) – nausea/diarrhea | Yes (3 resolved after stop) | Mild, self-limited; 1 pancreatitis case |
| *Lin et al.* | 2025 | 3297 | NA | NA | Not specified |
| *Liou et al.* | 2018 | 7 | Occasional mild nausea | None | Well tolerated |
| *Mahmoud et al.* | 2023 | 41 | Few mild GI symptoms | None | No discontinuations |
| *Mahzari et al.* | 2024 | 38 | Occasional GI intolerance | None | Improved with continuation |
| *Mallik et al.* | 2023 | 23 | NA | NA | Not specified |
| *Orandi et al.* | 2025 | 18016 | NA | NA | Not specified |
| *Sahi et al.* | 2025 | 77 | GI symptoms most common AE | None | Mostly mild; no serious events |
| *Sato et al.* | 2023 | 73 | NA | NA | Not specified |
| *Vigara et al.* | 2022 | 40 | NA | NA | Not specified |
| *Vigara et al.* | 2024 | 96 | 16 pts (16.7%) nausea/vomiting/diarrhea | 11 discontinued (11%) | 5 improved with dose reduction |
| *Zelada et al.* | 2025 | 25 | Mild GI symptoms | None | No pancreatitis or hospitalizations |

**Abbreviations:** GI, gastrointestinal; AE, adverse events; NA, not available.

**Supplementary Table S8.** Characteristics and key findings of abstracts and conference materials reporting GLP-1RA use in kidney transplant recipients

| **First author, year** | **Study design** | **Country** | **Population** | **Sample size** | **GLP-1RA**  **therapy** | **Follow-up (months)** | **Key efficacy findings** | **Renal outcomes** | **Safety outcomes** | **Limitations** |
| --- | --- | --- | --- | --- | --- | --- | --- | --- | --- | --- |
| Acosta et al. (2025) | RC | USA | KTR with pre-transplant T2DM | 18 | GLP-1RA alone (66.7%), GLP-1RA+SGLT2i (33.3%) | 12 | Improved HbA1c, BMI, fasting glucose, LDL cholesterol | Combination therapy improved eGFR | No discontinuations due to side effects; tacrolimus levels changed with combination therapy | Very small sample; retrospective |
| Attallah et al. (2022) | RC | UAE | KTR with pre-existing DM and PTDM | 28 | Semaglutide | 12 | HbA1c decreased by 1.2%; significant weight loss | Significant reduction in proteinuria; stable creatinine | 1 pancreatitis, 1 unstable angina; transient GI symptoms; no hypoglycemia | Small, single-center retrospective |
| Lim et al. (2025) | RC | USA | KTR with or without DM | 73 | Tirzepatide | 12 | Significant reductions in HbA1c and BMI | Stable creatinine and proteinuria | CNI dose adjustment required in ~34%; kidney function stable | Single center retrospective |
| Martin et al. (2025) | RC | USA | KTR with or without DM | 40 | Semaglutide, dulaglutide, liraglutide | 12 | No significant HbA1c or BMI change | Significant reduction in proteinuria; stable eGFR | Minimal safety events; well tolerated | Small sample; retrospective |
| Miura et al. (2025) | RC | Japan | KTR | 25 | Tirzepatide (n=19), liraglutide (n=6) | 6 | Improved HbA1c and significant weight loss with tirzepatide | Stable creatinine and proteinuria | No significant renal safety concerns | Very small sample; retrospective |
| Pham et al. (2025) | RC | USA | KTR with pre-existing or PTDM | 68 | GLP-1RA (unspecified) | 12 | Significant weight loss; no significant HbA1c change | Renal outcomes not reported | Tacrolimus levels stable; low MACE rate | Retrospective; small sample |
| Sridhar et al. (2023) | RC | Canada, Belgium | KTR with or without DM | 227 | GLP-1RA alone (34.8%), SGLT2i alone (34.3%), combination (30.8%) | 1 | No difference in CV outcomes or graft failure | Expected initial eGFR dip with SGLT2i | No increased infection risk; discontinuation rates 19–41% | Retrospective design |
|  |  |  |  |  |  |  |  |  |  |  |

**Abbreviations:** GLP-1RA, glucagon-like peptide-1 receptor agonist; KTR, kidney transplant recipient; PTDM, post-transplant diabetes mellitus; eGFR, estimated glomerular filtration rate; BMI, body mass index; CNI, calcineurin inhibitor; MACE, major adverse cardiovascular events; RC, retrospective cohort; UAE, United Arab Emirates; USA, United States of America.

**Supplementary Table S9.** Sensitivity of pooled estimates to meta-analytic model choice

| **Outcome** | **Number of studies (k)** | **Random-effects (REML) estimate, 95% CI, p** | **Fixed-effect estimate, 95% CI, p** | **Interpretation change (Y/N)** |
| --- | --- | --- | --- | --- |
| All-cause mortality (HR) | 4 | HR = 0.53, 95% CI 0.36–0.79, p = 0.002 | HR = 0.53, 95% CI 0.45 to 0.62, p < 0.00001 | No |
| All-cause mortality (OR) | 5 | OR = 0.23, 95% CI 0.11 to 0.48, p = 0.0001 | OR = 0.42, 95% CI 0.37 to 0.47, p < 0.00001 | No |
| MACE | 3 | OR = 0.55, 95% CI 0.47–0.66, p < 0.00001 | OR = 0.55, 95% CI 0.47–0.66, p < 0.00001 | No |
| eGFR 12 months (comparator) | 3 | MD = 4.52; 95% CI 0.11 to 8.92, p = 0.04 | MD = 2.43, 95% CI 2.21 to 2.65, p < 0.00001 | No |
| eGFR 3 months (within-group) | 3 | MD = 0.12 mL/min/1.73 m², 95% CI −3.41 to +3.66, p = 0.95 | MD = 1.46, 95% CI 0.09 to 2.84, p =0.04 | Yes |
| eGFR 6 months (within-group) | 5 | MD = 1.99 mL/min/1.73 m², 95% CI 0.52 to 3.47, p = 0.008 | MD = 1.99 mL/min/1.73 m², 95% CI 0.52 to 3.47, p = 0.008 | No |
| eGFR 12 months (within-group) | 6 | MD = 2.24 mL/min/1.73 m², 95% CI 0.02 to 4.46, p = 0.05 | MD = 1.84, 95% CI 0.48 to 3.20, p < 0.008 | No |
| eGFR 24 months (within-group) | 2 | MD = −0.06 mL/min/1.73 m², 95% CI −0.22 to 0.10, p = 0.44 | MD = −0.06 mL/min/1.73 m², 95% CI −0.22 to 0.10, p = 0.44 | No |
| HbA1c (comparator) | 5 | MD = −0.21%, 95% CI −0.51 to 0.08, p = 0.15 | MD = −0.04, 95% CI −0.12 to 0.03, p = 0.27 | No |
| HbA1c (within-group) | 13 | MD = −0.54%, 95% CI −0.89 to −0.19, p = 0.002 | MD = −0.00, 95% CI −0.06 to 0.05, p = 0.87 | Yes, but no small study number |
| BMI (comparator) | 4 | MD = −1.04 kg/m², 95% CI −1.95 to −0.14, p = 0.02 | MD = −0.70, 95% CI −0.98 to −0.42, p < 0.00001 | No |
| BMI (within-group) | 12 | SMD = −0.32, 95% CI −0.49 to −0.15, p = 0.0002 | SMD = −0.03, 95% CI −0.06 to −0.00, p = 0.04 | No |
| Tacrolimus 6 months (within-group) | 6 | MD = 0.08 ng/mL, 95% CI −0.35 to 0.19, p = 0.56 | MD = 0.08 ng/mL, 95% CI −0.35 to 0.19, p = 0.56 | No |
| Tacrolimus 12 months (within-group) | 2 | MD = −1.15 ng/mL, 95% CI −1.85 to −0.44, p = 0.001 | MD = −1.04, 95% CI −1.46 to −0.61, p < 0.00001 | No |
| Insulin need (within-group) | 4 | MD = −8.16 units of insulin, 95% CI -15.33 to -1.00, p = 0.03 | MD = −4.01, 95% CI −6.52 to −1.51, p = 0.002 | No |
| uACR (within-group) | 3 | MD = −26.67, 95% CI −41.31 to −12.04, p = 0.0004 | −26.67, 95% CI −41.3 to −12.04, p = 0.0004 | No |

**Supplementary Figure S1.** Funnel plots to assess publication bias (meta-analyses with ≥10 studies). (A) HbA1c within-group analysis. (B) BMI within-group analysis

**A.**

**B.**
